# Robust computational reconstitution – a new method for the comparative analysis of gene expression in tissues and isolated cell fractions

**DOI:** 10.1186/1471-2105-7-369

**Published:** 2006-08-04

**Authors:** Martin Hoffmann, Dirk Pohlers, Dirk Koczan, Hans-Jürgen Thiesen, Stefan Wölfl, Raimund W Kinne

**Affiliations:** 1 Leibniz Institute for Natural Products Research and Infection Biology – Hans Knöll Institute, Beutenbergstr. 11a, Jena, Germany; 2 Experimental Rheumatology Unit, Department of Orthopedics, Friedrich Schiller University Jena, Jena, Germany; 3 Institute of Immunology, University of Rostock, Rostock, Germany; 4 Department of Pharmacy and Molecular Biotechnology, Ruprecht Karls University Heidelberg, Heidelberg, Germany

## Abstract

**Background:**

Biological tissues consist of various cell types that differentially contribute to physiological and pathophysiological processes. Determining and analyzing cell type-specific gene expression under diverse conditions is therefore a central aim of biomedical research. The present study compares gene expression profiles in whole tissues and isolated cell fractions purified from these tissues in patients with rheumatoid arthritis and osteoarthritis.

**Results:**

The expression profiles of the whole tissues were compared to computationally reconstituted expression profiles that combine the expression profiles of the isolated cell fractions (macrophages, fibroblasts, and non-adherent cells) according to their relative mRNA proportions in the tissue. The mRNA proportions were determined by trimmed robust regression using only the most robustly-expressed genes (1/3 to 1/2 of all measured genes), i.e. those showing the most similar expression in tissue and isolated cell fractions. The relative mRNA proportions were determined using several different chip evaluation methods, among which the MAS 5.0 signal algorithm appeared to be most robust. The computed mRNA proportions agreed well with the cell proportions determined by immunohistochemistry except for a minor number of outliers. Genes that were either regulated (i.e. differentially-expressed in tissue and isolated cell fractions) or robustly-expressed in all patients were identified using different test statistics.

**Conclusion:**

Robust Computational Reconstitution uses an intermediate number of robustly-expressed genes to estimate the relative mRNA proportions. This avoids both the exclusive dependence on the robust expression of individual, highly cell type-specific marker genes and the bias towards an equal distribution upon inclusion of all genes for computation.

## Background

The comparative analysis of gene expression in diseased tissues and its isolated cell fractions can be used to identify genes with potential pathophysiological relevance, including those involved in interactions among different cell types. In the present study 'isolated cell fractions' are defined as cultivated cell populations of individual cell types purified from the respective tissue samples. A direct approach to the gene expression of specific cell types in the tissue is their microdissection from the tissue. Isolation and amplification of mRNA from microdissected single cells or pure cell type subpopulations has recently been established and described [1,2]. However, this method is just emerging, still having technical problems with reliable cell type markers, exact dissection, and representative mRNA extraction and amplification [3,4]. Therefore, instead of comparing gene expression profiles of individual cell types between tissue and isolated cell fractions, the present study compared the gene expression profiles of whole tissues and computationally reconstituted expression profiles that combine the expression profiles of the isolated cell fractions according to their relative mRNA proportions in the tissue. These relative mRNA proportions were determined using trimmed robust regression.

Methods for the reconstruction of cell type-specific expression profiles and relative proportions have already been proposed in the literature. The marker gene approach [5,6] determines the relative mRNA proportions from the expression of highly cell type-specific marker genes. A drawback of this method is its dependence on the robust expression of single genes. Venet et al. [7], Stuart et al. [8], and Lähdesmaeki et al. [9] identified cell type-specific expression profiles from tissue samples differing in their cell type composition. Venet et al. [7] and Lähdesmaeki et al. [9] computed the cell type-specific expression profiles and their corresponding relative proportions simultaneously (matrix factorization of the tissue gene expression matrix), whereas Stuart et al. [8] determined the cell proportions experimentally and then calculated the respective expression values (gene-wise regression). The method of Lu et al. [10] and the present study are different from the three previous approaches in that they use actually measured, cell type-specific expression profiles and determine the relative mRNA proportions computationally (tissue-wise regression). Whereas Lu et al. [10] compared desynchronized yeast cell 'tissues' and five isolated cell fractions consisting of synchronized yeast cells in the *G*_1_, *S*, *G*_2_, *M*, and *M*/*G*_1 _cell cycle phases, the present study compares synovial tissues with the isolated cell fractions of adherent macrophages, adherent fibroblasts. and non-adherent cells. The study of Lu et al. [10], however, did not address the relative importance of regulated gene expression resulting from the induction of cell cycle arrest. In contrast, the present study demonstrates for the first time that in order to avoid a bias towards an equal distribution of the computed mRNA proportions, as many regulated genes as possible must be excluded from the analysis. Here, 'regulated genes' are defined as showing a differential expression between tissue and isolated cell fractions, whereas 'robustly-expressed genes' show a similar expression under both conditions. Differential gene expression was also investigated in the study of Ghosh [11], in which the gene expression of tumor tissue samples was compared to that of normal tissue controls (probabilistic mixture model). This study accounted for the varying relative proportions of stromal tissue within the tumor samples and assumed the gene expression in tumor and normal tissue to be perfectly robust, i.e. independent of their relative proportions in the sample (as is also the case in the studies of Venet et al. [7]. Stuart et al. [8], Lähdesmaeki et al. [9], and Lu et al. [10]). Thus, differential expression in the study of Ghosh [11] refers to expression differences between tumor and normal tissue, whereas regulated expression in the present study refers to expression differences between two conditions (tissue versus isolated cell fractions).

Synovial membranes (inner aspect of the joint capsule) consist of three main cell types: macrophages and fibroblasts, both adhering to the culture vessel, and mixed non-adherent cells. These cell types are expected to contribute differentially to the pathogenesis of rheumatic diseases by expressing pro-inflammatory and pro-destructive genes. Therefore, gene expression profiles of the whole synovial tissue and the respective isolated cell fractions of patients with rheumatoid arthritis (RA) and osteoarthritis (OA) were analyzed using Affymetrix GeneChip technology. The performance of the newly developed reconstitution algorithm was validated using two mRNA mixing experiments performed by the authors and the mixing part of the GeneLogic dilution study [12]. The computed mRNA proportions were compared to the cell proportions determined by immunohistochemistry. Regulated and robustly-expressed genes selected according to statistical evidence and pathophysiological relevance are listed in the supplementary material.

## Results

**Figure 1:**
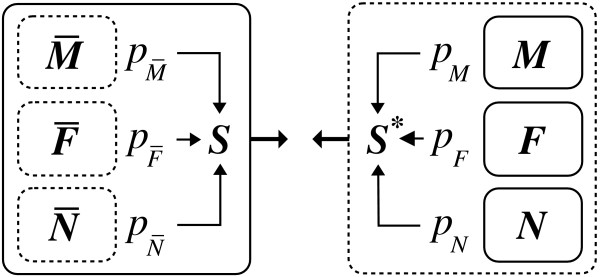
**Computational reconstitution**. Schematic view of the gene expression in the synovial tissue (left) and the computationally reconstituted tissue profile (right). The expression of the synovial tissue is composed of the expression of macrophages M¯
 MathType@MTEF@5@5@+=feaafiart1ev1aaatCvAUfKttLearuWrP9MDH5MBPbIqV92AaeXatLxBI9gBaebbnrfifHhDYfgasaacH8akY=wiFfYdH8Gipec8Eeeu0xXdbba9frFj0=OqFfea0dXdd9vqai=hGuQ8kuc9pgc9s8qqaq=dirpe0xb9q8qiLsFr0=vr0=vr0dc8meaabaqaciaacaGaaeqabaqabeGadaaakeaaieWacuWFnbqtgaqeaaaa@2DEF@, fibroblasts F¯
 MathType@MTEF@5@5@+=feaafiart1ev1aaatCvAUfKttLearuWrP9MDH5MBPbIqV92AaeXatLxBI9gBaebbnrfifHhDYfgasaacH8akY=wiFfYdH8Gipec8Eeeu0xXdbba9frFj0=OqFfea0dXdd9vqai=hGuQ8kuc9pgc9s8qqaq=dirpe0xb9q8qiLsFr0=vr0=vr0dc8meaabaqaciaacaGaaeqabaqabeGadaaakeaaieWacuWFgbGrgaqeaaaa@2DE1@, and non-adherent cells N¯
 MathType@MTEF@5@5@+=feaafiart1ev1aaatCvAUfKttLearuWrP9MDH5MBPbIqV92AaeXatLxBI9gBaebbnrfifHhDYfgasaacH8akY=wiFfYdH8Gipec8Eeeu0xXdbba9frFj0=OqFfea0dXdd9vqai=hGuQ8kuc9pgc9s8qqaq=dirpe0xb9q8qiLsFr0=vr0=vr0dc8meaabaqaciaacaGaaeqabaqabeGadaaakeaaieWacuWFobGtgaqeaaaa@2DF1@. The expression of the whole tissue is measured as the overall expression profile ***S***. The computationally reconstituted tissue profile S* is composed of the measured expression profiles of the isolated cell fractions, i.e. of macrophages ***M***, fibroblasts ***F***, and non-adherent cells ***N***. The relative mRNA proportions *p*_*M*_, *p*_*F*_, and *p*_*N *_are determined by matching the computationally reconstituted profile S* with the measured tissue profile ***S***, The measured expression profiles are enframed using solid lines.

The relative mRNA proportions of macrophages (*p*_*M*_), fibroblasts (*p*_*F*_), and non-adherent cells (*p*_*N*_) were determined for each tissue sample by matching the measured synovial tissue expression profile ***S ***and the computationally reconstituted expression profile S* = *p*_*M *_***M ***+ *p*_*F *_***F ***+ *p*_*N *_***N***, which in turn combines the measured expression profiles of the isolated cell fractions of macrophages (***M***), fibroblasts (***F***), and non-adherent cells (***N***) according to their relative mRNA proportions (Figure 1).

The matching of ***S ***and S* as a function of the relative mRNA proportions was performed using trimmed robust regression (Methods section, subsection Mathematical model). Trimmed regression only uses part of the data in order to exclude outliers that otherwise would bias the result. Here, the result is given by the set of relative mRNA proportions that minimize the differences between ***S ***and S* as quantified by the regression objective function. Solutions to trimmed regression problems are in general not unique due to the data subset choice, however, the solutions generally become more alike with increasing subset size. In the present study, the trimmed regression problem was solved for an increasing number of included genes. For each such number the optimization routine was initialized from several different starting values. The standard deviation of the results obtained from these random initializations was used to assess the similarity of the solutions. The relative mRNA proportions were determined from the respective ensemble means at the minimum number of included genes, for which the ensemble standard deviations approached zero, i.e. for which the solution was almost unique. This 'educated guess' (heuristic) approach accounts for the fact that the solution is completely indeterminate towards zero included genes and increasingly biased towards an equal distribution upon inclusion of all genes (Methods section, subsection Mathematical model). The general performance of this methodology is demonstrated in this first subsection. In order to assess the effect of data preprocessing on the present results the influence of different chip evaluation methods was investigated (Different chip evaluation methods subsection). The preliminary knowledge of the true relative mRNA proportions in mRNA mixing experiments was used to validate the present methodology as well as the chip evaluation methods (Mixing experiments subsection). In addition, the computed mRNA proportions were compared to the cell proportions determined by immunohistochemistry and those obtained using the marker gene approach (Immunohistochemistry and marker genes subsection). Finally, genes that were regulated and robustly-expressed in all patients were identified using different test statistics (Regulated and robustly-expressed genes subsection).

### General performance

**Figure 2:**
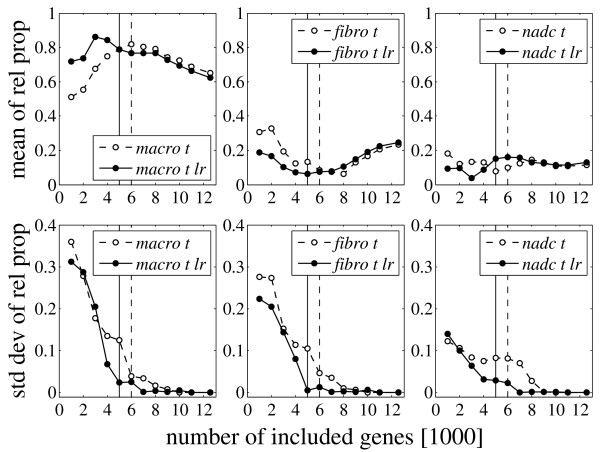
**Results for patient 2**. Mean and standard deviation of the computed relative mRNA proportions for macrophages (*macro*), fibroblasts (*fibro*) and non-adherent cells (*nadc*) for patient 2 as a function of the number *k *of genes included in the trimmed regression approach (Methods section, subsection Mathematical model). The measured expression profiles are MAS-S probe set summaries normalized by a symmetric trimmed mean (*t*) (Methods section, subsection Data preparation). The reconstituted tissue profile is either not normalized (dashed) or normalized by stepwise local regression (*lr*, solid) (Methods section, Data preparation). The proportions are determined from the respective means at the number of genes for which the standard deviations approach zero. The chosen numbers are indicated by the dashed and solid (*lr*) vertical lines, respectively. The convergence is faster for additional local regression (*lr*), but the resulting proportions are similar. The mRNA proportions are estimated to be *p*_*M *_= 0.82/0.79 (*lr*) for macrophages, *p*_*F *_= 0.09/0.06 (*lr*) for fibroblasts and *p*_*N *_= 0.10/0.15 (*lr*) for non-adherent cells. These values were determined at 6000 and 5000 (*lr*) included genes, respectively.

The proposed method for the determination of the relative mRNA proportions is demonstrated using the data of patient 2. Figure 2 shows how the means and standard deviations of the computed relative mRNA proportions of macrophages (*p*_*M*_), fibroblasts (*p*_*F*_), and non-adherent cells (*p*_*N*_) depend on the number *k *of genes included in the trimmed regression approach (Methods section, subsection Mathematical model). The mean is variable for small *k*. settles to a constant value for intermediate *k *and shows a slow and incomplete convergence towards an equal distribution (*p*_*M *_= *p*_*F *_= *p*_*N *_= 1/3) for large *k*. The respective standard deviations approach zero at an intermediate number of included genes. This number was used to determine the mRNA proportions from the respective mean values. The solid curves correspond to data, for which an additional local regression normalization was performed for the measured and reconstituted tissue profiles in each algorithmic step (Methods section, subsection Data preparation). The decrease of the standard deviation is faster for additional local regression normalization in this case, but the resulting mRNA proportions are similar.

The general curve progression in Figure 2 is similar to that obtained from a probabilistic model described in Supplementary Section A (Supplementary Figure A.2, see Additional file 1). However, the best estimate of the 'true' tissue mRNA proportions assumed by the model was obtained for the smallest number of included genes (in contrast to an intermediate number in Figure 2). This difference can be attributed to the striking roughness and overall flatness of the empirical objective function (3) used for trimmed regression (Methods section, subsection Mathematical model; Supplementary Figure A.3, see Additional file 1), if only a small number of genes is included. Hence, the means of the computed relative proportions converge to an equal distribution towards both ends. For increasing *k*, this is due to the inclusion of more and more regulated genes with expression levels non-correlated or negatively correlated between tissue and isolated cell fractions (Supplementary Section A.2, see Additional file 1). For decreasing *k*, this is due to the increasing roughness (increasing number of local minima) and overall flatness of the objective function (Methods section, subsection Mathematical model; Supplementary Section A.3, see Additional file 1). The relative mRNA proportions of the tissue can be estimated from the respective means of the computed mRNA proportions, as soon as the respective standard deviations become sufficiently small. The distribution of the individual mRNA proportions can then be assumed to be symmetric, implying the equality of mean and global minimum [13]. The drop of the standard deviation was indeed the most important criterion, but the curve progression and the agreement between the curves for the two different chip normalization methods (with and without local regression) were also taken into account.

### Different chip evaluation methods

There is an ongoing discussion about the question which probe set summary and which chip normalization method proves optimal for evaluating oligonucleotide microarrays. For assessing the effect of different chip evaluation methods on the results obtained by Robust Computational Reconstitution, four different probe set summaries were applied: MAS-S, MAS-C [14,15], RMA [16], and MBEI [17] (Methods section, subsection Data preparation). In addition, four different normalization methods were tested for the MAS-S and MAS-C summaries: trimmed mean only (*t*) [14,15], trimmed mean plus cyclic local regression (*clr*) [18], quantile normalization (*q*) [18,19], or centralization (*c*) [20] (Methods section, subsection Data preparation). The results are summarized in Table 1.

The computed mRNA fractions vary considerably among different probe set summaries and, for MAS-S and MAS-C, among different normalization methods. The methodological scatter differed among patients. It was quantified in terms of the pooled Mean Absolute Deviation (MAD) of the relative proportions across methods calculated with regard to the respective mean values, i.e. MAD = ∑C∑m|pC(m)−p¯C|/|m||C|
 MathType@MTEF@5@5@+=feaafiart1ev1aaatCvAUfKttLearuWrP9MDH5MBPbIqV92AaeXatLxBI9gBaebbnrfifHhDYfgasaacH8akY=wiFfYdH8Gipec8Eeeu0xXdbba9frFj0=OqFfea0dXdd9vqai=hGuQ8kuc9pgc9s8qqaq=dirpe0xb9q8qiLsFr0=vr0=vr0dc8meaabaqaciaacaGaaeqabaqabeGadaaakeaadaaeqaqaamaaqababaGaeiiFaWNaemiCaa3aa0baaSqaaiabdoeadbqaaiabcIcaOiabd2gaTjabcMcaPaaaaeaacqWGTbqBaeqaniabggHiLdaaleaacqWGdbWqaeqaniabggHiLdGccqGHsislcuWGWbaCgaqeamaaBaaaleaacqWGdbWqaeqaaOGaeiiFaWNaei4la8IaeiiFaWNaemyBa0MaeiiFaWNaeiiFaWNaem4qamKaeiiFaWhaaa@48A8@, in which p¯C=∑mpC(m)/|m|
 MathType@MTEF@5@5@+=feaafiart1ev1aaatCvAUfKttLearuWrP9MDH5MBPbIqV92AaeXatLxBI9gBaebbnrfifHhDYfgasaacH8akY=wiFfYdH8Gipec8Eeeu0xXdbba9frFj0=OqFfea0dXdd9vqai=hGuQ8kuc9pgc9s8qqaq=dirpe0xb9q8qiLsFr0=vr0=vr0dc8meaabaqaciaacaGaaeqabaqabeGadaaakeaacuWGWbaCgaqeamaaBaaaleaacqWGdbWqaeqaaOGaeyypa0ZaaabeaeaacqWGWbaCdaqhaaWcbaGaem4qameabaGaeiikaGIaemyBa0MaeiykaKcaaaqaaiabd2gaTbqab0GaeyyeIuoakiabc+caViabcYha8jabd2gaTjabcYha8baa@3EC0@ denotes the mean across methods (*m*) for a given cell type (*C*) (Table 2). The hybridization for patient 1 was repeated two times. Using the original tissue chip (HG-U95A), an MAD of 15% (in absolute value) was calculated. It was reduced to 5% and 9% using the second and third tissue chip replicate (HG-U95Av2), respectively (Table 2). Considering only the four different chip normalization methods applied to the MAS-S summaries resulted in a lower MAD for all patients, except for patient 2. Additional stepwise local regression normalization (*lr*) had little effect when all methods were considered, however, the MAD was greatly reduced by *lr *for the MAS-S normalization methods (all within 1–3%, Table 2).

The MAS-S summaries were preferred to the MAS-C, RMA, and MBEI summaries in this study because the agreement between the curves with and without stepwise local regression normalization (*lr*) was generally better for the MAS-S summaries (Figure 2 and Supplementary Figures C.1 and C.2, see Additional file 1). In addition. RMA, MBEI, and MAS-C tended to more extreme proportions close to 100% for patients 2, 5, and 6 (Table 1 and Supplementary Table C.1, see Additional file 1). In view of the relative cell proportions determined by immunohistochemistry (see subsection Immunohistochemistry and marker genes), the less extreme values obtained from MAS-S are more plausible. Moreover, the use of MAS-C resulted in irregular curves for patient 1 (original tissue chip, Supplementary Figure C.2, see Additional file 1).

The results obtained by excluding weakly-expressed genes (either < 100 or < 200 signal intensity) were within the range of the values listed in Table 1. This was also true for data filtering by Affymetrix present calls and transformation of the data according to the variance stabilization method of Huber et al. [21,22] (this is presumably due to the fact that the data are already corrected by MAS 5.0 in order to avoid negative intensities). Masking of outliers as performed by the MAS 5.0 software also did not alter the results. This suggests that measurement errors associated with weakly-expressed genes, outliers, or saturation of certain probe sets play a minor role for the estimation of the relative mRNA proportions using trimmed robust regression.

The effect of the experimental chip quality was assessed by correlating the MAD of the computed mRNA fractions (Table 2) with several relevant chip operating figures provided by the Affymetrix 'Expression Report' (Supplementary Section D, Supplementary Figures D.1 and D.2, see Additional file 1). Generally, the MAD was positively correlated with the noise and background level of the chips. However, due to the small number of patients the results strongly depended on individual patients (inhomogeneity correlation), in particular on patient 1 (original tissue chip).

### Mixing experiments

In mixing experiments, mRNA samples from different sources are mixed together in defined proportions and subsequently hybridized to chips. These experiments were used to validate the present computational approach. Two mixing experiments were performed by the authors with material from patient 2. The other experimental data were derived from the mixing part of the GeneLogic dilution study [12].

**Figure 3:**
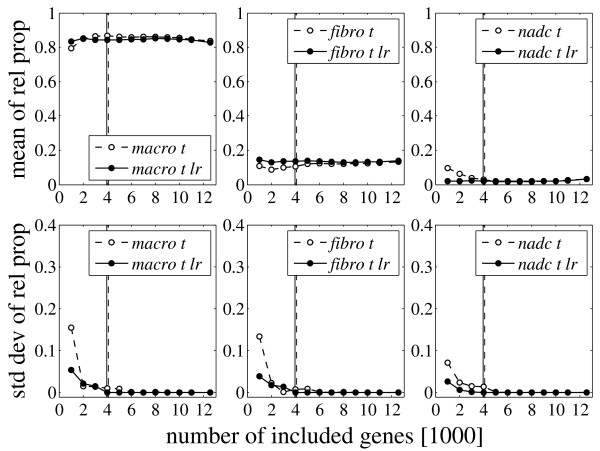
**Results for mixing experiment 2**. Mean and standard deviation of the computed mRNA proportions of macrophages (*macro*), fibroblasts (*fibro*) and non-adherent cells (*nadc*) for the second mixing experiment as a function of the number of included genes. This experiment corresponds to Figure 2, except that the tissue expression profile was not obtained by preparing mRNA from the whole synovial tissue, but by mixing mRNA samples from the isolated cell fractions according to the relative proportions *p*_*M *_= 0.86, *p*_*F *_= 0.11, and *p*_*N *_= 0.03. These proportions were almost perfectly determined when no further normalization of the reconstituted tissue profile was performed (dashed curves): *p*_*M *_= 0.86, *p*_*F *_= 0.10, *p*_*N *_= 0.04. Applying additional stepwise normalization (*lr*) (solid curves) resulted in *p*_*M *_= 0.85, *p*_*F *_= 0.13, *p*_*N *_= 0.02. All proportions were determined at 4000 included genes.

The target values for the mRNA proportions of the first two mixing experiments were set according to the range of values computed for patient 2 (Table 1). Figure 3 shows the results for the second mixing experiment using trimmed mean normalized MAS-S probe set summaries. The proportions are virtually fixed from the beginning and the standard deviations drop at only a few thousand included genes. This picture was typical for all mixing experiments, probe set summaries, and chip normalization methods of the present study. The number of included genes, at which the computed mRNA proportions were determined, was between 2000 and 4000 in all cases.

Table 3 summarizes the results in terms of the pooled MAD of the relative mRNA proportions, calculated with regard to the respective target values. All methods gave better results for the second mixing experiment. This difference cannot be explained by the experimental quality, because both chips had very similar operating figures. MAS-C tended to perform somewhat better than MAS-S, and both MAS-S and MAS-C gave better results than RMA, MBEI, and MBEI-GP. In contrast to MAS-S and MAS-C, the results for RMA, MBEI, and MBEI-GP were improved (with one exception) by additional stepwise local regression normalization (*lr*).

The GeneLogic dilution study contains 25 chips, in which mRNA samples from liver and SNB19 cells were mixed for SNB-proportions of 0. 0.25, 0.50. 0.75. and 1. and subsequently hybridized to HG-U95Av2 GeneChips. Replicas of these 5 mixes were processed by 5 different scanners resulting in a total of 25 experiments.

**Figure 4:**
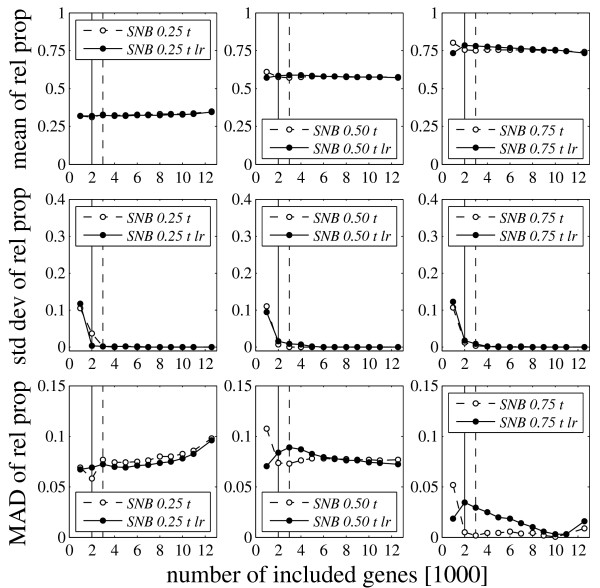
**Results for the GeneLogic Dilution Study**. Mean, standard deviation, and MAD of the computed mRNA proportions with respect to the target SNB-proportions of 0.25, 0.5 and 0.75. The displayed values are averages across 5 replicas. The results were calculated using trimmed mean (*t*) normalized MAS-S probe set summaries. Additional normalization by stepwise local regression is indicated by *lr *(solid curves). The proportions were determined at 3000 and 2000 (*lr*) included genes.

Figure 4 shows mean, standard deviation, and MAD of the computed mRNA proportions for SNB-proportions of 0.25, 0.5, and 0.75. The MADs were calculated with respect to these nominal target values. The standard deviations drop very early and the proportions were generally determined at 2000 included genes, with very few exceptions. The MAD of mixes 25 and 50 is quite high (roughly 4–10%) for all chip evaluation methods possibly indicating some imprecision in sample weighing. The results summarized in Table 4 show that, as in the mixing experiments of the authors, MAS-C tended to perform somewhat better than MAS-S (except for some cases in mix 75). The results for RMA and MBEI are similar to those for the MAS summaries.

### Immunohistochemistry and marker genes

Immunohistochemical staining was used to assess the cellular composition in the synovial tissue samples. The obtained cell type proportions of macrophages (pMc
 MathType@MTEF@5@5@+=feaafiart1ev1aaatCvAUfKttLearuWrP9MDH5MBPbIqV92AaeXatLxBI9gBaebbnrfifHhDYfgasaacH8akY=wiFfYdH8Gipec8Eeeu0xXdbba9frFj0=OqFfea0dXdd9vqai=hGuQ8kuc9pgc9s8qqaq=dirpe0xb9q8qiLsFr0=vr0=vr0dc8meaabaqaciaacaGaaeqabaqabeGadaaakeaacqWGWbaCdaqhaaWcbaGaemyta0eabaGaem4yamgaaaaa@30B4@), fibroblasts (pFc
 MathType@MTEF@5@5@+=feaafiart1ev1aaatCvAUfKttLearuWrP9MDH5MBPbIqV92AaeXatLxBI9gBaebbnrfifHhDYfgasaacH8akY=wiFfYdH8Gipec8Eeeu0xXdbba9frFj0=OqFfea0dXdd9vqai=hGuQ8kuc9pgc9s8qqaq=dirpe0xb9q8qiLsFr0=vr0=vr0dc8meaabaqaciaacaGaaeqabaqabeGadaaakeaacqWGWbaCdaqhaaWcbaGaemOrayeabaGaem4yamgaaaaa@30A6@), and non-adherent cells (pNc
 MathType@MTEF@5@5@+=feaafiart1ev1aaatCvAUfKttLearuWrP9MDH5MBPbIqV92AaeXatLxBI9gBaebbnrfifHhDYfgasaacH8akY=wiFfYdH8Gipec8Eeeu0xXdbba9frFj0=OqFfea0dXdd9vqai=hGuQ8kuc9pgc9s8qqaq=dirpe0xb9q8qiLsFr0=vr0=vr0dc8meaabaqaciaacaGaaeqabaqabeGadaaakeaacqWGWbaCdaqhaaWcbaGaemOta4eabaGaem4yamgaaaaa@30B6@) (Table 5) were compared to the respective mRNA proportions (*p*_*M*_, *p*_*F *_and *p*_*N*_) as determined by Robust Computational Reconstitution. For the comparison, each cell type *C *was assumed to contain a specific amount of mRNA *r*_*C*_. The mRNA proportions of the model thus read

p^C(pc)=rCpCc∑C¯rC¯pC¯c with pc=(pMc,pFc,pNc).     (1)
 MathType@MTEF@5@5@+=feaafiart1ev1aaatCvAUfKttLearuWrP9MDH5MBPbIqV92AaeXatLxBI9gBamXvP5wqSXMqHnxAJn0BKvguHDwzZbqegyvzYrwyUfgarqqtubsr4rNCHbGeaGqiA8vkIkVAFgIELiFeLkFeLk=iY=Hhbbf9v8qqaqFr0xc9pk0xbba9q8WqFfeaY=biLkVcLq=JHqVepeea0=as0db9vqpepesP0xe9Fve9Fve9GapdbaqaaeGacaGaaiaabeqaamqadiabaaGcbaGafmiCaaNbaKaadaWgaaWcbaGaem4qameabeaakiabcIcaOGWadiaa=bhadaahaaWcbeqaaiabdogaJbaakiabcMcaPiabg2da9maalaaabaGaemOCai3aaSbaaSqaaiabdoeadbqabaGccqWGWbaCdaqhaaWcbaGaem4qameabaGaem4yamgaaaGcbaWaaabeaeaacqWGYbGCdaWgaaWcbaGafm4qamKbaebaaeqaaaqaaiqbdoeadzaaraaabeqdcqGHris5aOGaemiCaa3aa0baaSqaaiqbdoeadzaaraaabaGaem4yamgaaaaakiabbccaGiabbEha3jabbMgaPjabbsha0jabbIgaOjabbccaGiaa=bhadaahaaWcbeqaaiabdogaJbaakiabg2da9maabmGabaGaemiCaa3aa0baaSqaaiabd2eanbqaaiabdogaJbaakiabcYcaSiabdchaWnaaDaaaleaacqWGgbGraeaacqWGJbWyaaGccqGGSaalcqWGWbaCdaqhaaWcbaGaemOta4eabaGaem4yamgaaaGccaGLOaGaayzkaaGaeiOla4IaaCzcaiaaxMaadaqadiqaaiabigdaXaGaayjkaiaawMcaaaaa@743C@

**Figure 5:**
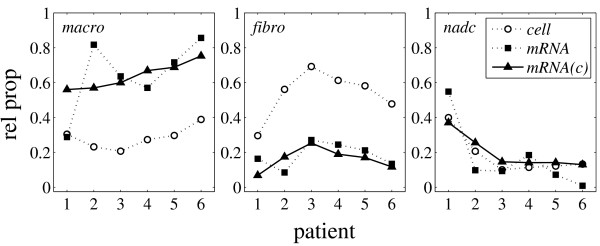
**Comparison of relative cell and mRNA proportions**. Relative cell proportions determined by immunohistochemistry (open circles; Table 5), relative mRNA proportions calculated by Robust Computational Reconstitution (solid squares; Table 1, MAS-S *t*), and relative mRNA proportions according to model equation (1) (solid triangles; fit to the computed relative mRNA proportions). The square root of the coefficient of determination is *R *= 0.90. The regression p-value is *p *= 10^-6 ^(least squares regression of 18 values using 2 parameters). The fitted model mRNA proportions are similar to the cell proportions multiplied by a constant factor reflecting the respective cellular mRNA content. Part of the large apparent differences in cellular mRNA content can possibly be attributed to differential mRNA yield due to experimental methodology, like mRNA extraction and amplification, or differential specificities of the cell type markers used in immunohistochemistry.

The model effectively contains two independent parameters that were fitted by matching the mRNA proportions of the model p^
 MathType@MTEF@5@5@+=feaafiart1ev1aaatCvAUfKttLearuWrP9MDH5MBPbIqV92AaeXatLxBI9gBaebbnrfifHhDYfgasaacH8akY=wiFfYdH8Gipec8Eeeu0xXdbba9frFj0=OqFfea0dXdd9vqai=hGuQ8kuc9pgc9s8qqaq=dirpe0xb9q8qiLsFr0=vr0=vr0dc8meaabaqaciaacaGaaeqabaqabeGadaaakeaacuWGWbaCgaqcaaaa@2E25@_*C *_(***p***^*c*^) to the 18 mRNA proportions (6 patients, 3 proportions each) computed by Robust Computational Reconstitution using least squares regression (Table 1, MAS-S *t*; Figure 5). The computed mRNA and cell proportions of non-adherent cells agreed almost one-to-one. Hence, the mRNA content of macrophages *r*_*M *_and fibroblasts *r*_*F *_were assessed relative to *r*_*N*_. Macrophages appeared to contain four times as much mRNA as non-adherent cells (*r*_*M *_= 3.98 *r*_*N*_), whereas fibroblasts seemed to contain only a quarter of it (*r*_*F *_= 0.25 *r*_*N*_). Excluding patient 1 from the analysis somewhat improved the fit for patients 2 to 6 (data not shown) but the resulting ratios for macrophages (*r*_*M *_= 4.56 *r*_*N*_) and fibroblasts (*r*_*F *_= 0.57 *r*_*N*_) were qualitatively similar. Part of the large apparent differences in mRNA content between cell types can possibly be attributed to differential mRNA yield due to experimental methodology, like mRNA extraction and amplification [3,23]. For example, de Bruin et al. [3] observed a 3.5-fold higher mRNA yield for tumor compared to stroma cells. In addition, differential specificities of the cell type markers used in immunohistochemistry may result in over- or underestimation of the relative cell proportions. The assumption that the mRNA content *r*_*C *_is independent of composition results from the usage of robustly-expressed genes for calculating the relative mRNA proportions. Nevertheless, the fit to the computed mRNA proportions was somewhat improved by assuming the mRNA content to depend on composition (linear ansatz; data not shown). Although this result suggests a possible dependence of the cellular mRNA content on the cell type composition, data overfitting by inclusion of too many adjustable parameters has to be avoided.

Cell type-specific marker genes allow to determine the mRNA proportions from the ratio of the expression in the synovial tissue (*s*_*i*_) and the respective isolated cell fraction (e.g., *p*_*M *_= *s*_*i*_/*m*_*i *_for macrophages) provided the marker gene (*i*) is very specific (no expression in fibroblasts and non-adherent cells) and robust (same expression in tissue and isolated cell fractions). In the present study, the relative mRNA proportions calculated using Robust Computational Reconstitution and marker genes showed a markedly higher than random consistency only for fibroblasts (Supplementary Table F.1, see Additional file 1). Obviously, the two premises required by the marker gene approach, specificity and robustness, are not fulfilled simultaneously, at least not for macrophages and non-adherent cells (the former being known to be quite responsive to stimulation and the latter being subject to a confounding heterogeneity of their cell type composition).

### Regulated and robustly-expressed genes

Regulated and robustly-expressed genes were identified using six different test statistics and log-transformed expression values of the measured (*S*) and reconstituted (S*) tissues of patients 1 to 6. The first four methods are well established techniques in the post-genomic era: Significance Analysis of Microarrays (SAM) [24], one-sample paired t-test (t-test1), homoscedastic two-sample t-test (t-test2) (being equivalent to one-way ANOVA for two groups), and VERAandSAM (V&S) [25]. In addition, two new criteria, *μ*-test and MAD-test, were introduced in the present study owing to the fact that SAM, t-test1, and t-test2 tended to select robustly-expressed genes with low expression strength (Supplementary Figure C.2, see Additional file 1). This bias is a result of the fact that the standard error appears in the denominator of these test statistics and that weakly-expressed genes generally show a higher standard error than strongly-expressed genes (log-transformed data). As a consequence, the newly-defined *μ*- and MAD-test statistics, *t*_*μ *_= *μ*_1 _- *μ*_2 _= mean(*s *- s*) and *t*_MAD _= mean(|*s *- s*|), respectively. (*s *= log *S*, s* = log S*), do not contain the standard error. The *μ*-test, and also V&S, appeared to be almost unbiased with respect to expression strength. The MAD-test (most strongly related to the regression objective function (3) in the Methods section, subsection Mathematical model) showed a clear preference for highly-expressed genes when applied to identify robustly-expressed genes (Supplementary Figure G.2, see Additional file 1). This may reflect the fact that in general small differences in gene expression are observed for highly-expressed genes (well known from so called M-A-plots for log-transformed expression data). Also, highly-expressed genes serving fundamental roles in the cell are commonly used as housekeeping genes [23]. The p-values of t-test1 and t-test2 were calculated using both the Student distribution and the permutation method described in Storey and Tibshirani [26]. For the paired t-test1 *n*_*P *_= 1000 permutations were used. For the unpaired t-test2 all *n*_*P *_= (6 out of 12)/2 = 462 possibilities for obtaining different absolute values of the test statistic were enumerated. The Pearson correlation coefficient between the respective p-values was *c*_1 _= 0.999979 for t-test1 and *c*_2 _= 0.999986 for t-test2 suggesting that the gene expression data of the present study are roughly log-normally distributed. Consequently, the Student distribution derived p-values were used for t-test1 and t-test2. The p-values of SAM and MAD-test were calculated using the permutation method and *n*_*p *_= 1000 permutations, those of *μ*-test by enumerating all *n*_*p *_= 462 possibilities. The λ-values of V&S are based on an expression level-dependent error model [25]. The pairwise correlations between the six statistical methods are shown in Supplementary Figure G.1 (see Additional file 1). The best correspondence was observed among SAM, t-test1, t-test2, and *μ*-test (Pearson correlation between 0.844 and 0.977). The MAD-test differed most from all other methods (Pearson correlation between 0.240 and 0.677). Also, this test statistic was the only one showing a two-modal p-value distribution in contrast to the uni-modal distributions assumed by the method of Storey and Tibshirani [26] for calculating False Discovery Rate (FDR)-related q-values. The other methods allowed to estimate the proportion of null-hypothesis genes quite consistently (between 0.5 and 0.6). Regulated and robustly-expressed genes were identified by 1.) applying each of the six individual test statistics independently and 2.) selecting top-ranking genes with respect to pathophysiological relevance (Supplementary Section G, Supplementary Tables G.2 and G.3, see Additional file 1). A total of 62 regulated and 48 robustly-expressed genes were selected. Each method contributed some selected genes and the approach of combining different complementary test statistics for gene selection, as proposed in the present study, may prove useful in future investigations.

## Discussion

Determining and analyzing the gene expression profiles of different tissue cell types under diverse physiological and pathophysiological conditions is a central aim of biomedical research. However, microdissection of single cells or pure cell types, as well as mRNA isolation and amplification [1,2] still bears basic technical problems [3,4]. Therefore, gene expression profiles of whole tissue samples and purified cell types were compared in the present study. Using Robust Computational Reconstitution, the required mRNA proportions and the set of robustly-expressed genes that allow for their determination were simultaneously identified.

The present results suggest that MAS-S provided the most stable probe set summaries. This is in contrast to the study of Irizarry et al. [16], but is in line with the results of Choe et al. [27]. This discrepancy may be due to the data sets used. Whereas in the study of Irizarry et al. [16] only few spiked-in genes were present, Choe et al. [27] spiked-in more than 1300 genes. Real world applications like the present study possibly contain more biological and technical noise and the subtraction of the mismatch probes from the perfect-match probes (MAS 5.0) may be appropriate in this case. Recent detailed sequence level studies of Binder and Preibisch [28,29] resulted in similar conclusions. However, when Shedden et al. [30] applied their FDR-based pairwise comparison method to ovary and colon tumor data, MAS 5.0 performed inferior to most other methods.

Chip-wise (non-linear) averaging of the probes before comparison to other chips (MAS-S) may also be less susceptible to the effects of outliers than the initial comparison of probes between different chips and subsequent averaging of the differences (MAS-C). There were only few differences in the present study among the different chip normalization methods applied to the MAS-S and MAS-C summaries. However, the application of quantile normalization and also centralization resulted in more outliers compared to the remaining normalization methods. Quantile normalization also tended to perform suboptimally without additional local regression normalization in the study of Choe et al. [27].

The accuracy of Robust Computational Reconstitution depends on the fraction of robustly-expressed genes, the quality of experimentation, and the appropriateness of the chip evaluation method. The variance is estimated to be in the range of 5–10% in absolute value based on the variability of the computed mRNA proportions among methods (Table 2) and the expected bias introduced by the imperfect robustness of even the most robustly-expressed genes (Supplementary Figures A.1 and A.2, see Additional file 1). A sensitivity analysis (resampling of relative proportions) showed that the rank order of differentially-expressed genes is only moderately affected by this variance. However, the maximum shift in rank order was always large indicating that some differentially-expressed genes may be lost even from a moderately extended list of top-ranking genes. This maximum rank shift was close to that of random permutations but the third quartile of the rank shift distribution was already much smaller than that observed for random permutations (rank shift of 60 compared to 9300 for the 200 top-ranking genes and a resampling standard deviation of 5%).

The results for patient 1 largely differed from all other patients. On the one hand this may be due to the fact that HG-U95A chips were used instead of HG-U95Av2 chips. On the other hand patient 1 is the only type I RA patient (patients 2 and 3 are type II RA) [31,32]. This was confirmed by immunohistochemistry and comparison with synovial tissue gene expression profiles of 12 other classified RA patients (using the distance to the respective expression means and Affymetrix' best-matches probe set list for matching HG-U133A and HG-U95Av2 chips). Hence, the differences between RA patient 1 and RA patients 2 and 3 are substantial.

Remarkably, the computed mRNA proportions considerably differ from the cell proportions determined by immunohistochemistry (Figure 5). Most striking is the prevalence of macrophage mRNA over fibroblast mRNA in contrast to the dominance of fibroblasts over macrophages in the cell composition, implying that macrophages produce 8 to 16 times as much mRNA as do fibroblasts. The results for the mRNA proportions may be biased because the isolated fractions of macrophages and non-adherent cells were not perfectly pure with respect to fibroblasts (resulting in apparently higher mRNA proportions for macrophages). Also, the mRNA yield of different cell types may be severalfold different due to experimental methodology, e.g. mRNA extraction and amplification [3,23]. Furthermore, the cell proportions as determined by immunohistochemistry may be somewhat imprecise due to the limited specificity of this semi-quantitative method. Nevertheless, the augmented mRNA production of macrophages compared to fibroblasts is expected to be still valid under idealized experimental conditions. The central aim addressed by the present study was the cell type-specific gene expression in synovial tissue and its dependence on cell type composition (purified cell fractions representing an extreme case of composition). Cell type-specific gene expression was also addressed by Venet et al. [7], who assumed the individual expression profiles to be largely uncorrelated. According to our own-experience (the correlation coefficients among the isolated cell fractions showed values as high as 0.7 and 0.9), this is an arguable assumption, as also admitted by Venet et al. [7] themselves. Lähdesmaeki et al. [9] used different constraints in their least squares approach. Similar to Venet et al. [7] and Lu et al. [10] and in contrast to the present study, they included all genes in the sum of squares and showed this to be appropriate in the case of mixing experiments (as also demonstrated in the present study). However, their method, as well as the methods of Venet et al. and Lu et al. (which is otherwise equivalent to the present approach), will give biased results in the presence of regulated (i.e. composition-dependent) gene expression. Using an intermediate number of robustly-expressed genes (present study) avoids, on the one hand, the exclusive dependence on the robust expression of individual highly cell type-specific marker genes and, on the other hand, the bias towards an equal distribution when including all genes in matrix factorization or regression. This statement remains valid despite the inability of all current *in silico *microdissection methods (including our own; Methods section, subsection Mathematical model and Supplementary Section H, see Additional file 1) to directly assign composition-dependent changes in gene expression to specific cell types.

## Conclusion

The proposed method of Robust Computational Reconstitution is applicable if there is a sufficient number of robustly-expressed genes whose expression is highly correlated between tissue and isolated cell fractions. The existence of such housekeeping genes is plausible and well-known [23] since many genes code for basic cell functions that must be maintained across different environmental conditions. The present approach improves previously published methods for the determination of the relative mRNA proportions of different cell types by the exclusion of regulated genes that bias the result towards an equal distribution. In addition, it can identify robustly-expressed genes (representing basic metabolism or persistent pathological changes) and responsive genes that change their expression between tissue and isolated cell fractions (possibly reflecting physiological or pathological cell communication processes). Both are of biomedical interest and can be further screened for pathophysiological relevance.

Evidently, microdissection of single cells or pure cell types [1,2] is a promising experimental technique. However, it is still under steady development and, for the time being, the preferential use of macrodissection in combination with *in silico *microdissection techniques has been recently recommended [3]. The method of the present study can thus be readily used as an effective research tool and a supporting reference method for further studies.

### Note added in proof

During the review process of the present manuscript Wang et al. [33] also published a related paper dealing with the cellular composition of complex tissues and the relative contribution of the individual cell types to tissue gene expression.

## Methods

### Mathematical model

The proposed method for determining the cell type-specific mRNA composition of synovial tissue samples (applicable to any other tissue) is based on a comparison between the measured expression profile *S *of the whole synovial tissue and the computationally reconstituted tissue profile

S* = *p*_*M *_***M ***+ *p*_*F *_***F ***+ *p*_*N *_***N***,     (2)

which is composed of the measured expression profiles of the isolated cell fractions, i.e. isolated adherent macrophages ***M***, adherent fibroblasts ***F***, and non-adherent cells ***N***, according to their respective computational mRNA proportions *p*_*M*_, *p*_*F*_, and *p*_*N *_(Figure 1).

The proposed computational approach aims at the simultaneous identification of the cell type-specific mRNA proportions and the particular set of robustly-expressed genes, which allows to reliably determine these proportions. This is achieved by minimizing the trimmed sum of absolute differences

Ds(k)=∑i∈Ik|si−si∗|     (3)
 MathType@MTEF@5@5@+=feaafiart1ev1aaatCvAUfKttLearuWrP9MDH5MBPbIqV92AaeXatLxBI9gBaebbnrfifHhDYfgasaacH8akY=wiFfYdH8Gipec8Eeeu0xXdbba9frFj0=OqFfea0dXdd9vqai=hGuQ8kuc9pgc9s8qqaq=dirpe0xb9q8qiLsFr0=vr0=vr0dc8meaabaqaciaacaGaaeqabaqabeGadaaakeaacqWGebardaqhaaWcbaGaem4CamhabaGaeiikaGIaem4AaSMaeiykaKcaaOGaeyypa0ZaaabuaeaacqGG8baFcqWGZbWCdaWgaaWcbaGaemyAaKgabeaakiabgkHiTiabdohaZnaaDaaaleaacqWGPbqAaeaacqGHxiIkaaGccqGG8baFaSqaaiabdMgaPjabgIGiolabdMeajnaaBaaameaacqWGRbWAaeqaaaWcbeqdcqGHris5aOGaaCzcaiaaxMaadaqadiqaaiabiodaZaGaayjkaiaawMcaaaaa@49D8@

between the gene expression in the measured and the reconstituted tissue with respect to *p*_*M *_and *p*_*F*_, treating *p*_*N *_= 1 - *p*_*M *_- *p*_*F *_as a dependent variable (equivalently, an optimization routine able of handling constraints could be used with all three variables). In Equation (3) *s*_*i *_= log *S*_*i *_and si∗
 MathType@MTEF@5@5@+=feaafiart1ev1aaatCvAUfKttLearuWrP9MDH5MBPbIqV92AaeXatLxBI9gBaebbnrfifHhDYfgasaacH8akY=wiFfYdH8Gipec8Eeeu0xXdbba9frFj0=OqFfea0dXdd9vqai=hGuQ8kuc9pgc9s8qqaq=dirpe0xb9q8qiLsFr0=vr0=vr0dc8meaabaqaciaacaGaaeqabaqabeGadaaakeaacqWGZbWCdaqhaaWcbaGaemyAaKgabaGaey4fIOcaaaaa@3092@ = log Si∗
 MathType@MTEF@5@5@+=feaafiart1ev1aaatCvAUfKttLearuWrP9MDH5MBPbIqV92AaeXatLxBI9gBaebbnrfifHhDYfgasaacH8akY=wiFfYdH8Gipec8Eeeu0xXdbba9frFj0=OqFfea0dXdd9vqai=hGuQ8kuc9pgc9s8qqaq=dirpe0xb9q8qiLsFr0=vr0=vr0dc8meaabaqaciaacaGaaeqabaqabeGadaaakeaacqWGtbWudaqhaaWcbaGaemyAaKgabaGaey4fIOcaaaaa@3052@ are log-transformed expression values in ***S ***and S*, respectively, and *k *denotes the number of genes included in the sum. For given values of *p*_*M *_and *p*_*F*_, the set of gene indices *I*_*k *_is determined in order to minimize the objective function Ds(k)
 MathType@MTEF@5@5@+=feaafiart1ev1aaatCvAUfKttLearuWrP9MDH5MBPbIqV92AaeXatLxBI9gBaebbnrfifHhDYfgasaacH8akY=wiFfYdH8Gipec8Eeeu0xXdbba9frFj0=OqFfea0dXdd9vqai=hGuQ8kuc9pgc9s8qqaq=dirpe0xb9q8qiLsFr0=vr0=vr0dc8meaabaqaciaacaGaaeqabaqabeGadaaakeaacqWGebardaqhaaWcbaGaem4CamhabaGaeiikaGIaem4AaSMaeiykaKcaaaaa@326A@. More specifically, the genes are sorted with respect to their absolute differences |*s*_*i *_- si∗
 MathType@MTEF@5@5@+=feaafiart1ev1aaatCvAUfKttLearuWrP9MDH5MBPbIqV92AaeXatLxBI9gBaebbnrfifHhDYfgasaacH8akY=wiFfYdH8Gipec8Eeeu0xXdbba9frFj0=OqFfea0dXdd9vqai=hGuQ8kuc9pgc9s8qqaq=dirpe0xb9q8qiLsFr0=vr0=vr0dc8meaabaqaciaacaGaaeqabaqabeGadaaakeaacqWGZbWCdaqhaaWcbaGaemyAaKgabaGaey4fIOcaaaaa@3092@|, in which si∗
 MathType@MTEF@5@5@+=feaafiart1ev1aaatCvAUfKttLearuWrP9MDH5MBPbIqV92AaeXatLxBI9gBaebbnrfifHhDYfgasaacH8akY=wiFfYdH8Gipec8Eeeu0xXdbba9frFj0=OqFfea0dXdd9vqai=hGuQ8kuc9pgc9s8qqaq=dirpe0xb9q8qiLsFr0=vr0=vr0dc8meaabaqaciaacaGaaeqabaqabeGadaaakeaacqWGZbWCdaqhaaWcbaGaemyAaKgabaGaey4fIOcaaaaa@3092@ = si∗
 MathType@MTEF@5@5@+=feaafiart1ev1aaatCvAUfKttLearuWrP9MDH5MBPbIqV92AaeXatLxBI9gBaebbnrfifHhDYfgasaacH8akY=wiFfYdH8Gipec8Eeeu0xXdbba9frFj0=OqFfea0dXdd9vqai=hGuQ8kuc9pgc9s8qqaq=dirpe0xb9q8qiLsFr0=vr0=vr0dc8meaabaqaciaacaGaaeqabaqabeGadaaakeaacqWGZbWCdaqhaaWcbaGaemyAaKgabaGaey4fIOcaaaaa@3092@(*p*_*M*_, *p*_*F*_) depends on the current values of *p*_*M *_and *p*_*F*_. Subsequently, the first *k *differences are summed up. Clearly, the proportions uniquely determine the rank order of the genes, whereas *k *determines how many of the sorted differences (genes) are used to calculate the objective function value. The described method is a trimmed least modulus (*L*_1_) regression. It is similar to the trimmed least squares (*L*_2_) regression described in [22] and [34], but is considered to be more robust with respect to y-outliers (however, graphs of the *L*_1 _and *L*_2 _objective functions as a function of *p*_*M *_and *p*_*F *_looked alike and *L*_1_- and *L*_2_-regression gave similar results; Figure 2 and Supplementary Figures A.3 and A.4. see Additional file 1). If only a small number of genes is included in the sum, the optimization routine (simplex algorithm) is likely to end up in a local minimum that depends on the starting values for *p*_*M *_and *p*_*F*_. This is because the trimmed sum Ds(k)
 MathType@MTEF@5@5@+=feaafiart1ev1aaatCvAUfKttLearuWrP9MDH5MBPbIqV92AaeXatLxBI9gBaebbnrfifHhDYfgasaacH8akY=wiFfYdH8Gipec8Eeeu0xXdbba9frFj0=OqFfea0dXdd9vqai=hGuQ8kuc9pgc9s8qqaq=dirpe0xb9q8qiLsFr0=vr0=vr0dc8meaabaqaciaacaGaaeqabaqabeGadaaakeaacqWGebardaqhaaWcbaGaem4CamhabaGaeiikaGIaem4AaSMaeiykaKcaaaaa@326A@ is very similar for different proportions, if the included genes can be chosen from a much larger set of genes providing a great variety of suitable expression values (resulting in a vast number of local minima for the present data; Supplementary Figure A.3, see Additional file 1). Increasing the number of included genes results in more unique values for the computed proportions (reduced number of local minima, Supplementary Figure A.3, see Additional file 1). However, the more regulated genes are included in the sum, the more the proportions will be biased towards an equal distribution (*p*_*M *_= *p*_*F *_= *p*_*N *_= 1/3) (Supplementary Section A, see Additional file 1). For this reason, the present study suggests to determine the mRNA proportions as soon as the standard deviations of the computed proportions approach zero, indicating the emergence of an unique global minimum and thus an unequivocal solution to the regression problem. The following simple algorithmic protocol was developed for the computation of the mRNA proportions (Table 6):

The results obtained from a statistical model for the expectation value ℰ
 MathType@MTEF@5@5@+=feaafiart1ev1aaatCvAUfKttLearuWrP9MDH5MBPbIqV92AaeXatLxBI9gBamrtHrhAL1wy0L2yHvtyaeHbnfgDOvwBHrxAJfwnaebbnrfifHhDYfgasaacH8akY=wiFfYdH8Gipec8Eeeu0xXdbba9frFj0=OqFfea0dXdd9vqai=hGuQ8kuc9pgc9s8qqaq=dirpe0xb9q8qiLsFr0=vr0=vr0dc8meaabaqaciaacaGaaeqabaWaaeGaeaaakeaaimaacqWFWesraaa@3785@[Ds(k)
 MathType@MTEF@5@5@+=feaafiart1ev1aaatCvAUfKttLearuWrP9MDH5MBPbIqV92AaeXatLxBI9gBaebbnrfifHhDYfgasaacH8akY=wiFfYdH8Gipec8Eeeu0xXdbba9frFj0=OqFfea0dXdd9vqai=hGuQ8kuc9pgc9s8qqaq=dirpe0xb9q8qiLsFr0=vr0=vr0dc8meaabaqaciaacaGaaeqabaqabeGadaaakeaacqWGebardaqhaaWcbaGaem4CamhabaGaeiikaGIaem4AaSMaeiykaKcaaaaa@326A@] of the objective function in Equation (3) compare well with those for the patient data (Figure 2 and Supplementary Figure A.2, see Additional file 1). The statistical model assumes normal distributed expression values. This is consistent with the fact that the data appear to be roughly log-normally distributed as evidenced by the high Pearson correlation (> 0.99997) between the t-test p-values determined by the Student distribution on the one hand and those p-values calculated according to the permutational method of Storey and Tibshirani [26] on the other hand (Results section, subsection Regulated and robustly-expressed genes). The good general correspondence between the patient data and the statistical model corroborates the suitability of the proposed method for estimating the relative mRNA proportions.

The differences *s*_*i *_- si∗
 MathType@MTEF@5@5@+=feaafiart1ev1aaatCvAUfKttLearuWrP9MDH5MBPbIqV92AaeXatLxBI9gBaebbnrfifHhDYfgasaacH8akY=wiFfYdH8Gipec8Eeeu0xXdbba9frFj0=OqFfea0dXdd9vqai=hGuQ8kuc9pgc9s8qqaq=dirpe0xb9q8qiLsFr0=vr0=vr0dc8meaabaqaciaacaGaaeqabaqabeGadaaakeaacqWGZbWCdaqhaaWcbaGaemyAaKgabaGaey4fIOcaaaaa@3092@ in gene expression between the whole tissue and the isolated cell fractions presumably reflect transcriptional changes due to different environmental conditions and it is desirable to know how each individual cell type is actually responding to this change. However, the cell type-specific changes in gene expression can only be determined by either actually measuring the gene expression of each cell type in the tissue (using e.g. microdissection) or by applying appropriate statistical estimates. Again, by using a normal distribution model (Supplementary Section H, see Additional file 1) and results obtained from the present study (Supplementary Table H.1, see Additional file 1) it is demonstrated that the distributional parameters of the model cannot be reliably estimated based on the present gene expression data. The inability to assign cell type-specific changes in gene expression was also recognized in the study of Stuart et al. [8], in which non-linear regression analysis revealed interactions between different cell types. The suggestion of Stuart et al. [8] to attribute the observed expression change to the cell type that expresses the respective gene most strongly is, however, not necessarily appropriate in all cases, e.g. for robustly-expressed marker genes [5].

### Experiments

Synovial tissue samples were obtained from 3 RA [35] and 3 OA patients [36] and prepared as previously described [31]. During the primary cell culture, **non-adherent cells **were removed by medium exchange on day 1. After 7 days, **synovial fibroblasts **were purified by removal of **macrophages **using anti-CD 14 magneto-beads. Total RNA was isolated and labeled according to the supplier's (Qiagen) instructions. Gene expression was analyzed using Affymetrix HG-U95A (patient 1) and HG-U95Av2 (patient 2–6) chips. The synovial tissue chip of patient 1 showed about twice as much noise as the other chips. Therefore, the hybridization was repeated twice (using HG-U95Av2 chips). Finally, the first repeat was selected for analysis. Two mixing experiments were performed, in which mRNA preparations of the isolated cell fractions of patient 2 were mixed according to two representative sets of relative mRNA proportions and subsequently hybridized to HG-U95Av2 chips. Immunohistochemical analysis was performed on cryostat sections of synovial membranes using marker antibodies for specific cell types. For each patient, the cellular composition was assessed for one to three different synovial tissue sections from samples adjacent to those, from which the mRNA was prepared (Table 5). Further details of the experimental materials and methods are given in the Supplementary Sections B and E (see Additional file 1).

### Data preparation

Microarrays were evaluated using four different probe set summaries: 1) single chip (MAS-S); and 2) chip comparison (MAS-C) algorithm of Affymetrix' Microarray Suite 5.0 [14,15]; 3) Robust Multi-Array Analysis (RMA) developed by Irizarry et al. [16]; and 4) Model Based Expression Index (MBEI) of Li and Wong [17] as implemented in R and the GenePublisher web-interface [37]. In addition, the MAS-S and MAS-C summaries were normalized using four different globalization methods: i) 2%-symmetric trimmed mean (*t*) [14,15]; ii) cyclic local regression (*clr*) [18]; iii) quantile normalization (*q*) [18,19]; and iv) centralization (*c*) [20]. The computationally reconstituted tissue profile S* was either not further normalized or normalized by local regression. This method is based on so called *M*-*A*-plots and normalizes two expression profiles, in this case *S *and S*, at the same time. The normalization has to be done in each algorithmic step, i.e. for every pair of relative proportions that is explored by the optimization algorithm. It was chosen to be a 200-genes symmetric moving average instead of the widely used loess procedure [18,38] in order to achieve reasonable computation times. The symmetric moving average was also used for the cyclic local regression normalization (*clr*). Centralization (*c*) requires the selection of a set of robustly-expressed genes that is used for normalization. This set was chosen to consist of those 1000 genes, for which at least one pairwise log-ratio was among the best conserved (i.e. showed the lowest mean absolute deviation) across the four chips per patient. The additive nature of Equation (2) requires absolute expression values. However, the chip comparison algorithm (MAS-C) computes log-ratios that compare a baseline experiment to different other experiments in a pairwise manner. The absolute expression values were reconstructed from the baseline experiment (*S*) and the respective log-ratios. The number of random starting values for the relative mRNA proportions (cf. algorithmic protocol. Table 6) was *l *= 25 throughout this study. The missing *N*-profile of patient 3 was substituted from patient 2 (both Type II RA) [32]. When HG-U95A and HG-U95Av2 chips were investigated simultaneously, the analysis was restricted to the 12533 probe sets common to both chips (excluding controls). For the RMA and MBEI summaries of patient 1 (tissue repeats) the mixture CDF environment provided by Bolstad [39] was used.

## Authors' contributions

MH developed mathematical methods, performed computations, and wrote the manuscript; DP conducted the experimental work and contributed to the manuscript; DK hybridized chips and contributed to the discussion, HJT and SW contributed to the design of the study and the discussion; RWK contributed to experimental work, discussion, and writing of the manuscript. All authors read and approved the final manuscript.

## Supplementary Material

Additional File 1 Supplementary Material. Self-contained pdf-file providing supplementary information. Supplementary sections A – H. Click here for file

## Figures and Tables

**Table 1:** Computed relative mRNA proportions. Computed relative mRNA proportions *p*_*M *_(macrophages) and *p*_*F *_(fibroblasts) for patients 1–6 calculated for different chip evaluation methods. The proportion of non-adherent cells is *p*_*N *_= 1 - *p*_*M *_- *p*_*F*_. The primed 1 in 'patient 1' (RA)' indicates the use of the second tissue chip replicate for patient 1. Additional stepwise local regression normalization is indicated by *lr*. The proportions are given in percent. Probe set summaries: MAS-S: Microarray Suite 5.0 signal algorithm, RMA: Robust Multiarray Analysis, MBEI: Model Based Expression Index, GP: GenePublisher (not available for HG-U95A chips, patient 1), the R implementation of MBEI was used with the same settings as in GP. Normalization methods: *t*: trimmed mean, *clr*: cyclic local regression, *q*: quantile normalization, *c*: centralization.

chip evaluation method	patient 1' (RA)				patient 2 (RA)				patient 3 (RA)				patient 4 (QA)				patient 5 (QA)				patient 6 (QA)			
	
			*lr*				*lr*				*lr*				*lr*				*lr*				*lr*	
	
	*M*	*F*	*M*	*F*	*M*	*F*	*M*	*F*	*M*	*F*	*M*	*F*	*M*	*F*	*M*	*F*	*M*	*F*	*M*	*F*	*M*	*F*	*M*	*F*
MAS-S *t*	29	16	29	19	82	9	79	6	64	27	64	29	57	24	57	27	72	21	69	20	86	13	81	19
MAS-S *t clr*	32	18	29	22	90	10	86	5	66	30	64	33	65	25	64	30	74	21	68	25	78	22	74	26
MAS-S *t q*	31	14	24	22	92	8	93	6	69	30	63	34	66	23	63	30	87	07	71	22	71	29	67	33
MAS-S *t c*	31	15	29	17	80	5	80	6	59	31	65	30	71	16	66	21	80	13	80	11	96	2	80	20
RMA	44	24	28	31	95	5	82	16	49	42	45	45	60	26	46	36	94	0	85	8	96	0	100	0
MBEI	44	15	42	19	91	5	76	2	38	52	45	49	66	2	57	10	98	0	92	5	100	0	99	1
MBEI-GP	--	--	--	--	98	1	88	9	26	62	43	55	57	5	38	18	92	0	94	0	100	0	100	0

**Table 2:** MAD of the computed mRNA proportions. Pooled mean absolute deviation (MAD) of the relative mRNA proportions across different chip evaluation methods, calculated with regard to the respective mean values. 'MAD all' refers to all seven methods: the four different normalizations of MAS-S, as well as RMA, MBEI, and MBEI-GP, whereas 'MAD MAS' only refers to the four different normalization methods of MAS-S. The additional application of stepwise local regression normalization is indicated by *lr*. The MAD refers to the proportions of Table 1 (given in percent).

patient	1	1'	1"	2	3	4	5	6
MAD all	15	5	9	4	9	7	6	7
MAD all *lr*	18	4	7	5	7	9	6	8
MAD MAS	7	1	2	4	3	3	4	6
MAD MAS *lr*	1	2	1	3	1	3	3	3

**Table 3:** MAD for mixing experiments 1 and 2. Pooled MAD of the computed relative mRNA proportions in mixing experiments 1 and 2 for different chip evaluation methods. The MAD is calculated with respect to the target values *p*_*M *_= 0.75, *p*_*F *_= 0.11, *p*_*N *_= 0.14 (mix 1) and *p*_*M *_= 0.86, *p*_*F *_= 0.11, *p*_*N *_= 0.03 (mix 2). 'MAS-S mean' and 'MAS-C mean' denote the means with respect to the four preceeding normalization methods. Notation according to Table 1.

Chip evaluation method	mix 1		mix 2	
	
		*lr*		*lr*
MAS-S *t*	1.2	2.2	0.8	1.3
MAS-S *t clr*	2.5	4.6	1.8	1.7
MAS-S *t q*	3.3	4.3	1.1	1.1
MAS-S *t c*	1.6	2.5	0.8	2.0
MAS-S mean	2.2	3.4	1.1	1.5
MAS-C *t*	0.6	1.9	0.5	1.1
MAS-C *t clr*	2.1	4.7	0.9	1.8
MAS-C *t q*	2.2	5.4	0.8	1.8
MAS-C *t c*	2.0	1.9	1.2	1.0
MAS-C mean	1.7	3.5	0.9	1.4
R.MA	9.7	6.8	2.6	2.9
MBEI	8.2	5.4	3.3	1.2
MBEI-GP	6.6	5.2	1.1	1.0

**Table 4:** MAD for the GeneLogic Dilution Study. Pooled MAD of the computed relative mRNA proportions in mixing experiments 25, 50, and 75 for different chip evaluation methods. The respective MAD is calculated with regard to the target values *p*_*S *_= 0.25, 0.50, and 0.75. Notation according to Table 3.

evaluation method	mix 25		mix 50		mix 75	
	
		*lr*		*lr*		*lr*
MAS-S *t*	7.7	6.9	7.3	8.4	0.2	3.4
MAS-S *t clr*	7.4	3.8	6.1	6.1	1.2	1.4
MAS-S *t q*	8.4	5.9	9.0	7.7	1.5	2.2
MAS-S *t c*	9.6	7.5	9.2	8.8	2.0	3.6
MAS-S mean	8.2	6.0	7.9	7.7	1.2	2.6
MAS-C *t*	6.0	5.7	6.2	7.2	2.0	2.2
MAS-C *t clr*	7.1	3.9	7.0	5.9	1.1	1.0
MAS-C *t q*	7.8	3.7	7.8	6.5	1.7	2.6
MAS-C *t c*	6.2	5.9	8.2	7.3	3.5	2.7
MAS-C mean	6.8	4.8	7.3	6.7	2.1	2.1
RMA	9.9	8.9	8.4	7.8	0.8	1.1
MBEI	8.0	8.2	8.1	7.5	1.7	1.0

**Table 5:** Cell proportions. Relative cell proportions of adherent macrophages *M*, adherent fibroblasts *F*, and non-adherent cells *N *for peitients 1–6 obtained from immunohistochemistry by analyzing tissue samples in close vicinity to the sample from which the mRNA was isolated. The fraction of non-adherent cells consists of T-cells (*T*), B-cells (*B*), plasma cells (*P*), and endothelial cells (*E*). If standard deviations are given, three samples were analyzed. The proportions are given in percent.

patient	1	2	3	4	5	6	mean
*M*	33 ± 14	22	19 ± 4	26 ± 3	29	39 ± 6	28
*F*	32 ± 17	54	63 ± 10	59 ± 16	57	48 ± 13	52
*N*	43 ± 22	20	9 ± 0	12 ± 11	12	13 ± 6	18
*T*	14 ± 10	0	0 ± 0	3 ± 5	3	7 ± 4	5
*B*	12 ± 13	0	0 ± 0	1 ± 2	0	1 ± 1	2
*P*	9 ± 2	0	0 ± 0	1 ± 2	0	2 ± 2	2
*E*	8 ± 2	20	9 ± 0	7 ± 4	9	3 ± 1	9

**Table 6:** Algorithmic Protocol

1) calculate the means and standard deviations of the computed mRNA proportions as a function of the number of included genes:
□ increase the number of included genes *k*
• generate *l *random starting values {pj0 MathType@MTEF@5@5@+=feaafiart1ev1aaatCvAUfKttLearuWrP9MDH5MBPbIqV92AaeXatLxBI9gBaebbnrfifHhDYfgasaacH8akY=wiFfYdH8Gipec8Eeeu0xXdbba9frFj0=OqFfea0dXdd9vqai=hGuQ8kuc9pgc9s8qqaq=dirpe0xb9q8qiLsFr0=vr0=vr0dc8meaabaqaciaacaGaaeqabaqabeGadaaakeaaieWacqWFWbaCdaqhaaWcbaGaemOAaOgabaGaeGimaadaaaaa@3095@} *j *= 1,..., *l*, in which pj0 MathType@MTEF@5@5@+=feaafiart1ev1aaatCvAUfKttLearuWrP9MDH5MBPbIqV92AaeXatLxBI9gBaebbnrfifHhDYfgasaacH8akY=wiFfYdH8Gipec8Eeeu0xXdbba9frFj0=OqFfea0dXdd9vqai=hGuQ8kuc9pgc9s8qqaq=dirpe0xb9q8qiLsFr0=vr0=vr0dc8meaabaqaciaacaGaaeqabaqabeGadaaakeaaieWacqWFWbaCdaqhaaWcbaGaemOAaOgabaGaeGimaadaaaaa@3095@ = (pMj0,pFj0,pNj0 MathType@MTEF@5@5@+=feaafiart1ev1aaatCvAUfKttLearuWrP9MDH5MBPbIqV92AaeXatLxBI9gBaebbnrfifHhDYfgasaacH8akY=wiFfYdH8Gipec8Eeeu0xXdbba9frFj0=OqFfea0dXdd9vqai=hGuQ8kuc9pgc9s8qqaq=dirpe0xb9q8qiLsFr0=vr0=vr0dc8meaabaqaciaacaGaaeqabaqabeGadaaakeaacqWGWbaCdaqhaaWcbaGaemyta0KaemOAaOgabaGaeGimaadaaOGaeiilaWIaemiCaa3aa0baaSqaaiabdAeagjabdQgaQbqaaiabicdaWaaakiabcYcaSiabdchaWnaaDaaaleaacqWGobGtcqWGQbGAaeaacqaIWaamaaaaaa@3D80@)
○ determine ***p***_*j *_= (*p*_*Mj*_, *p*_*Fj*_, *p*_*Nj*_) by minimizing the objective function Ds(k) MathType@MTEF@5@5@+=feaafiart1ev1aaatCvAUfKttLearuWrP9MDH5MBPbIqV92AaeXatLxBI9gBaebbnrfifHhDYfgasaacH8akY=wiFfYdH8Gipec8Eeeu0xXdbba9frFj0=OqFfea0dXdd9vqai=hGuQ8kuc9pgc9s8qqaq=dirpe0xb9q8qiLsFr0=vr0=vr0dc8meaabaqaciaacaGaaeqabaqabeGadaaakeaacqWGebardaqhaaWcbaGaem4CamhabaGaeiikaGIaem4AaSMaeiykaKcaaaaa@326A@ starting from pj0 MathType@MTEF@5@5@+=feaafiart1ev1aaatCvAUfKttLearuWrP9MDH5MBPbIqV92AaeXatLxBI9gBaebbnrfifHhDYfgasaacH8akY=wiFfYdH8Gipec8Eeeu0xXdbba9frFj0=OqFfea0dXdd9vqai=hGuQ8kuc9pgc9s8qqaq=dirpe0xb9q8qiLsFr0=vr0=vr0dc8meaabaqaciaacaGaaeqabaqabeGadaaakeaaieWacqWFWbaCdaqhaaWcbaGaemOAaOgabaGaeGimaadaaaaa@3095@
○ store ***p***_*j*_
• calculate the mean and standard deviation of {***p***_*j*_}*j *= 1,...,*l *for the current value of *k*
2) determine the means of the computed mRNA proportions at the smallest number of included genes, for which the standard deviations approach zero.

## References

[B1] Kamme F, Salunga R, Yu J, Tran DT, Zhu J, Luo L, Bittner A, Guo HQ, Miller N, Wan J, Erlander M (2003). Single-Cell Microarray Analysis in Hippocampus CA1: Demonstration and Validation of Cellular Heterogeneity. J Neurosci.

[B2] Taylor T, Nambiar P, Raja R, Cheung E, Rosenberg D, Anderegg B (2004). Microgenomics: Identification of New Expression Profiles Via Small and Single-Cell Sample Analyses. Cytometry A.

[B3] de Bruin E, van de Pas S, Lips E, van Eijk R, van der Zee M, Lombaerts M, van Wezel T, Marijnen C, van Krieken J, Medema J, van de Velde C, Eilers P, Peltenburg L (2005). Macrodissection versus microdissection of rectal carcinoma: minor influence of stroma cells to tumor cell gene expression profiles. BMC Genomics.

[B4] Wang H, Owens J, Shih J, Li M, Bonner R, Mushinski J (2006). Histological staining methods preparatory to laser capture microdissection significantly affect the integrity of the cellular RNA. BMC Genomics.

[B5] Schmid H, Henger A, Cohen C, Frach K, Gröne HJ, Schlöndorff D, Kretzler M (2003). Gene Expression Profiles of Podocyte-Associated Molecules as Diagnostic Markers in Acquired Proteinuric Diseases. J Am Soc Nephrol.

[B6] Häupl T, Grützkau A, Grün J, Kinne R, Berek C, Stuhlmüller B, Rohrlach T, Kaps C, Rudwaleit M, Morawietz L, Gursche A, Zacher J, Müller-Ladner U, Krenn V, Burmester GR, Radbruch A (2005). Dominant Role of B-cells and Monocytes in Rheumatoid Arthritis Based on Synovial Expression Profiles. American College of Rheumatology, 69th Annual Meeting, San Diego, CA, USA.

[B7] Venet D, Pecasse F, Maenhaut C, Bersini H (2001). Separation of samples into their constituents using gene expression data. Bioinformatics.

[B8] Stuart R, Wachsman W, Berry C, Wang-Rodriguez J, Wasserman L, Klacansky I, Masys D, Arden K, Goodison S, McClelland M, Wang Y, Sawyers A, Kalcheva I, Tarin D, Mercola D (2004). In silico dissection of cell-type-associated patterns of gene expression in prostate cancer. Proc Natl Acad Sci USA.

[B9] Lähdesmäki H, Shmulevich I, Dunmire V, Yli-Harja O, Zhang W (2005). In silico microdissection of microarray data from heterogeneous cell populations. BMC Bioinformatics.

[B10] Lu P, Nakorchevskiy A, Marcotte E (2003). Expression deconvolution: A reinterpretation of DNA microarray data reveals dynamic changes in cell populations. Proc Natl Acad Sci USA.

[B11] Ghosh D (2004). Mixture models for assessing differential expression in complex tissues using microarray data. Bioinformatics.

[B12] GeneLogic (2003). Dilution Study. http://www.genelogic.com.

[B13] Delyon B, Juditsky A, Benveniste A (1997). On the relationship between identification and local tests.

[B14] Liu WM, Mei R, Di X, Ryder T, Hubbell E, Dee S, Webster T, Harrington C, Ho MH, Baid J, Smeekens S (2002). Analysis of high density expression microarrays with signed-rank call algorithms. Bioinformatics.

[B15] Affymetrix (2002). Statistical Algorithms Reference Guide. http://www.affymetrix.com.

[B16] Irizarry R, Bolstad B, Collin F, Cope L, Hobbs B, Speed T (2003). Summaries of Affymetrix GeneChip probe level data. Nucleic Acids Res.

[B17] Li C, Wong W (2001). Model-based analysis of oligonucleotide arrays: Expression index computation and outlier detection. Proc Natl Acad Sci USA.

[B18] Bolstad B, Irizarry R, Astrand M, Speed T (2003). A comparison of normalization methods for high density oligonucleotide array data based on variance and bias. Bioinformatics.

[B19] Kroll T, Wölfl S (2002). Ranking: a closer look on globalisation methods for normalisation of gene expression arrays. Nucleic Acids Res.

[B20] Zien A, Aigner T, Zimmer R, Lengauer T (2001). Centralization: a new method for the normalization of gene expression data. Bioinformatics.

[B21] Huber W, von Heydebreck A, Sultmann H, Poustka A, Vingron M (2002). Variance stabilization applied to microarray data calibration and to the quantification of differential expression. Bioinformatics.

[B22] Huber W, von Heydebreck A, Sueltmann H, Poustka A, Vingron M (2003). Parameter estimation for the calibration and variance stabilization of microarray data. Stat Appl Genet Mol Biol.

[B23] Szabo A, Perou C, Karaca M, Perreard L, Quackenbush J, Bernard P (2004). Statistical modeling for selecting housekeeper genes. Genome Biol.

[B24] Tusher V, Tibshirani R, Chu G (2001). Significance analysis of microarrays applied to the ionizing radiation response. Proc Natl Acad Sci USA.

[B25] Ideker T, Thorsson V, Siegel A, Hood L (2000). Testing for Differentially-Expressed Genes by Maximum-Likelihood Analysis of Microarray Data. J Comput Biol.

[B26] Storey J, Tibshirani R (2003). Statistical significance for genomewide studies. Proc Natl Acad Sci USA.

[B27] Choe S, Boutros M, Michelson A, Church G, Halfon M (2005). Preferred analysis methods for Affymetrix GeneChips revealed by a wholly defined control dataset. Genome Biol.

[B28] Binder H (2006). Probing gene expression – sequence specific hybridization on microarrays. Bioinformatics of Genome Regulation and Structure II chap.

[B29] Binder H, Preibisch S (2006). GeneChip microarrays – signal intensities, RNA concentrations and probe sequences. J Phys: Condens Matter.

[B30] Shedden K, Chen W, Kuick R, Ghosh D, Macdonald J, Cho K, Giordano T, Gruber S, Fearon E, Taylor J, Hanash S (2005). Comparison of seven methods for producing Affymetrix expression scores based on False Discovery Rates in disease profiling data. BMC Bioinformatics.

[B31] Zimmermann T, Kunisch E, Pfeiffer R, Hirth A, Stahl H, Sack U, Laube A, Liesaus E, Roth A, Palombo-Kinne E, Emmrich F, Kinne R (2001). Isolation and characterization of rheumatoid arthritis synovial fibroblasts from primary culture – primary culture cells markedly differ from fourth-passage cells. Arthritis Res.

[B32] Ruschpler P, Stiehl P (2002). Shift in Th1 (IL-2 and IFN-gamma) and Th2 (IL-10 and IL-4) cytokine mRNA balance within two new histological main-types of rheumatoid-arthritis (RA). Cell Mol Biol.

[B33] Wang M, Master SR, Chodosh LA (2006). Computational expression deconvolution in a complex mammalian organ. BMC Bioinformatics.

[B34] Rousseeuw P, Leroy A (1987). Robust Regression and Outlier Detection.

[B35] Altman R, Asch E, Block D, Bole G, Borenstein D, Brandt K, Christy W, Cooke TD, Greenwald R, Hochberg Mea (1986). Development of criteria for the classification and reporting of osteoarthritis. Classification of osteoarthritis of the knee. Diagnostic and Therapeutic Criteria Committee of the American Rheumatism Association. Arthritis Rheum.

[B36] Arnett F, Edworthy S, Bloch D, McShane D, Fries J, Cooper N, Healey L, Kaplan S, Liang M, Luthra H (1988). The American Rheumatism Association 1987 revised criteria for the classification of rheumatoid arthritis. Arthritis Rheum.

[B37] Knudsen S, Workman C, Sicheritz-Ponten T, Friis C (2003). GenePublisher: Automated analysis of DNA microarray data. Nucleic Acids Res.

[B38] Dudoit S, Yang Y, Callow M, Speed T (2000). Statistical methods for identifying differentially expressed genes in replicated cDNA microarray experiments. Tech Rep 578, UC Berkeley, Division of Biostatistics.

[B39] Bolstad B (2004). Mixture CDF environments. http://bmbolstad.com/misc/mixtureCDF/MixtureCDF.html.

