# Supplementary material for: Robust computational reconstitution – a new method for the comparative analysis of gene expression in tissues and isolated cell fractions
Source: BMC Bioinformatics. 2006 Aug 4;7:369. doi: 10.1186/1471-2105-7-369 (PMC1574358; doi:10.1186/1471-2105-7-369)
Supplement: Additional File 1 — Supplementary Material. Self-contained pdf-file providing supplementary information. Supplementary sections A – H. [file 1471-2105-7-369-S1.pdf]

Supplementary Material

A Probabilistic Model

The objective of the first part of this section is to calculate the expectation value $\mathcal{E}[\delta_s]$ of the difference $\delta_s = s - s^*$ between the gene expression in the measured and reconstituted tissue and to show that the computational proportions that minimize $\mathcal{E}[\delta_s]$ converge to the true relative mRNA proportions in the tissue for strongly correlated genes and to an equal distribution ($p_M = p_F = p_N = 1/3$) for uncorrelated genes. In the second part, a model for Robust Computational Reconstitution is developed. This is done by calculating the expectation value $\mathcal{E}[D_s^{(k)}]$ of the trimmed sum $D_s^{(k)}$ in Equation (2) of the main text (using an empirically determined distribution of correlation coefficients) and determining the relative proportions that minimize $\mathcal{E}[D_s^{(k)}]$. In addition, the standard deviations of the relative proportions are calculated from the inverse of the second derivatives of $\mathcal{E}[D_s^{(k)}]$ [1]. The good correspondence to the numerical results for the patient data shown in Figure 2 of the main text corroborates the suitability of the proposed method for the determination of the relative mRNA proportions. The third part of this section includes graphs of the empirical objective function $D_s^{(k)}$ (trimmed sum) illustrating its dependence on the number of included genes k.

A.1 Convergence of the relative proportions

The model used for showing the convergence of the computed relative mRNA proportions is strongly simplified in that the difference $\delta_s = \log S - \log S^*$ (with $S = p_{\bar{M}}\,\bar{M} + p_{\bar{F}}\,\bar{F} + p_{\bar{N}}\,\bar{N}$ and $S^* = p_M\,M + p_F\,F + p_N\,N$) is approximated by

$$\delta_s = (p_{\bar{M}}\,\bar{m} - p_M\,m) + \left(p_{\bar{F}}\,\bar{f} - p_F\,f\right) + (p_{\bar{N}}\,\bar{n} - p_N\,n) \quad , \tag{A.1}$$

in which $\bar{m} = \log \bar{M}$, $m = \log M$, etc. This approximation was necessary because the full form of δ_s was not amenable to an analytical treatment. It can be justified in a statistical sense in that the measured log-transformed expression profiles s, m, f and n are all approximately normally distributed, which is also true for a linear combination of normally distributed random variables. The problem was further simplified in that all auto-correlation coefficients (e.g., $r_{\bar{m}m}$) were set equal to r, all cross-correlation coefficients (e.g., r_{mf}) were neglected, and all standard deviations were assumed to be equal to σ, because only the principal mechanisms were of interest. Under these assumptions, the difference δ_s is normally distributed with the standard deviation [2, 3]

$$\sigma_s = \sqrt{\sigma_m^2 + \sigma_f^2 + \sigma_n^2} \quad , \quad \text{in which e.g.,} \quad \sigma_m^2 = \left(p_{\bar{M}}^2 + p_M^2 - 2\,r\,p_{\bar{M}}\,p_M\right)\sigma^2 \quad . \tag{A.2}$$

Minimizing σ_s with respect to p_M, p_F, and p_M taking account of $p_M + p_F + p_M = 1$ results in

$$p_M = \left(p_{\bar{M}} - \frac{1}{3}\right) r + \frac{1}{3} \tag{A.3}$$

and analogous expressions for p_M and p_F. Hence, $(p_M, p_F, p_M) = (p_{\bar{M}}, p_{\bar{F}}, p_{\bar{N}})$ if the genes are fully correlated between synovial tissue and isolated cell fractions ($r = 1$) and $p_M = p_F = p_M = 1/3$ if they are independent ($r = 0$).

A.2 Model for Robust Computational Reconstitution

A realistic model for Robust Computational Reconstitution must allow for the fact that the correlation coefficient r is generally different for each gene. This was done by multiplying the Gaussian probability density function (PDF) of δ_s having standard deviation according to equation (A.2) (and thus a specific value for r) by an empirically determined piecewise linear PDF for the correlation coefficient r (Figure A.1) and subsequent marginalization with respect to r. The resulting modified PDF for δ_s was then used for calculating the expectation values $\mathcal{E}[D_s^{(k)}]$ of the trimmed sum $D_s^{(k)}$ in Equation (2) of the main text. This was done by summing up the expectation values of the lowest k values of the absolute difference $\rho = |\delta_s|$ in a random experiment with $\mathcal{N}$ (number of genes) trials. The expectation values for these k lowest values were calculated using order statistics [4, 5]. Let $F(\rho)$ be the cumulative distribution function (CDF) of ρ. Then, the CDF of the ith smallest value ρ_i (ith order statistic) is given by

$$F_i(\rho_i) = \sum_{j=i}^{\mathcal{N}} \binom{\mathcal{N}}{j} F(\rho_i)^j \left(1 - F(\rho_i)\right)^{\mathcal{N}-j} \quad , \quad \text{with} \quad \binom{\mathcal{N}}{j} = \frac{\mathcal{N}!}{j!\,(\mathcal{N}-j)!} \quad . \tag{A.4}$$

Using one partial integration, the expectation value of ρ_i was calculated according to

$$\mathcal{E}[\rho_i] = \int_0^\infty \rho\; f_i(\rho)\; d\rho = \bar{\rho} - \int_0^{\bar{\rho}} F_i(\rho)\; d\rho \quad , \tag{A.5}$$

with $f_i(\rho_i)$ denoting the PDF of ρ_i and $\bar{\rho}$ being an appropriate cut-off value, which was determined according to $1 - F(\bar{\rho})^{\mathcal{N}} < 10^{-8}$. The expectation value of $D_s^{(k)} = \sum_{i=1}^{k} \rho_i$ was then calculated by summing up the first k expectation values $\mathcal{E}[\rho_i]$. Finally, the relative proportions p_M, p_F, and p_N that minimize $\mathcal{E}[D_s^{(k)}]$ were calculated as a function of the number of included genes k. The results shown in Figure A.2 are similar to the numerical results obtained for the experimental data of patient 2 (Figure 2 of the main text) but give the best estimates for the relative proportions at the lowest number of included genes. This is because the expectation value $\mathcal{E}[D_s^{(k)}]$ is a smooth function of the proportions and the minimum can accurately be determined. For a specific data set, however, a multitude of equivalent local minima is scattered across the whole domain (triangle $0 \leq p_M + p_F \leq 1$) implying an equal distribution of the relative proportions. Small changes in the PDF of the correlation coefficient r near $r = 1$ have a strong influence on the maximum attainable precision of the estimated relative proportions. This is demonstrated by the differences between the dashed and the solid curves in Figures A.1 and A.2.

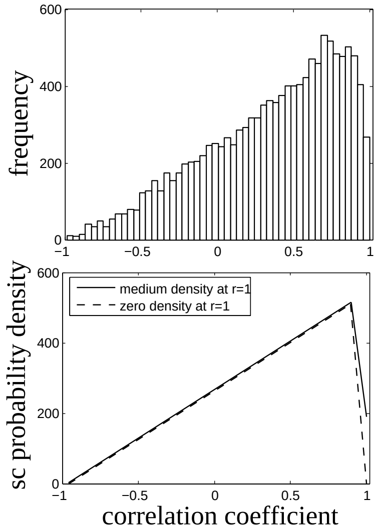

Figure A.1: Histogram for the correlation coefficient r between $s = \log S$ and $s^* = \log S^*$ (top) and the piecewise linear probability density function (PDF) used in the model (bottom, scaled to match the histogram). Solid line: medium density assumed at $r = 1$, dashed line: zero density assumed at $r = 1$.

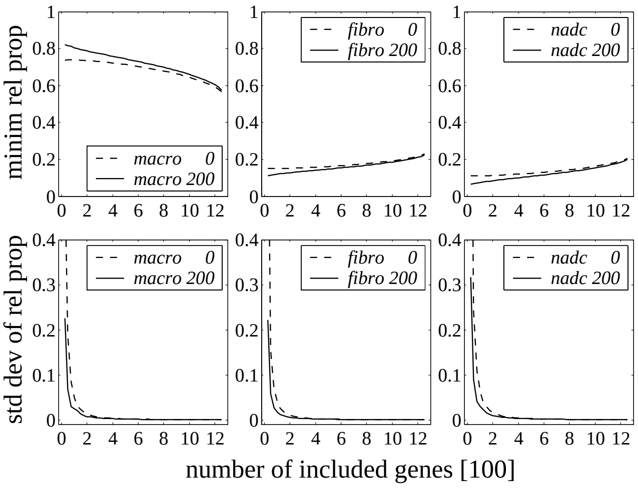

Figure A.2: Minimizing relative proportions of the expectation value $\mathcal{E}[D_s^{(k)}]$ for macrophages ($macro$), fibroblasts ($fibro$), and non-adherent cells ($nadc$) as a function of the number k of genes included in the trimmed sum $D_s^{(k)}$ (top) and the corresponding standard deviations estimated from the inverse of the second derivatives of $\mathcal{E}[D_s^{(k)}]$ [1] (bottom). Assumed true mRNA proportions in the tissue: $p_{\bar{M}} = 0.85, p_{\bar{F}} = 0.10$, and $p_{\bar{N}} = 0.05$. The curves are similar to those shown in Figure 2 of the main text, however, they do not converge towards an equal distribution for a small number of included genes because the expectation value $\mathcal{E}[D_s^{(k)}]$ is a smooth function of p_M and p_F (see text). The standard deviations drop earlier for the same reason. Solid line: medium density assumed at $r = 1$, dashed line: zero density assumed at $r = 1$ (see Figure A.1). The number of included genes ranges between 25 and 1253 (one 10th of the 12533 genes on the chips) because summation and integration according to Equations (A.4) and (A.5), respectively, was done numerically.

A.3 Empirical objective function of trimmed regression

In the present study the trimmed sum $D_s^{(k)}$ in Equation (2) of the main text is used as the objective function for determining the relative mRNA proportions of the tissue samples of each patient. The figures shown in this section illustrate the dependence of $D_s^{(k)}$ on the number of included genes k, which is essential to the method of robust computational reconstitution.

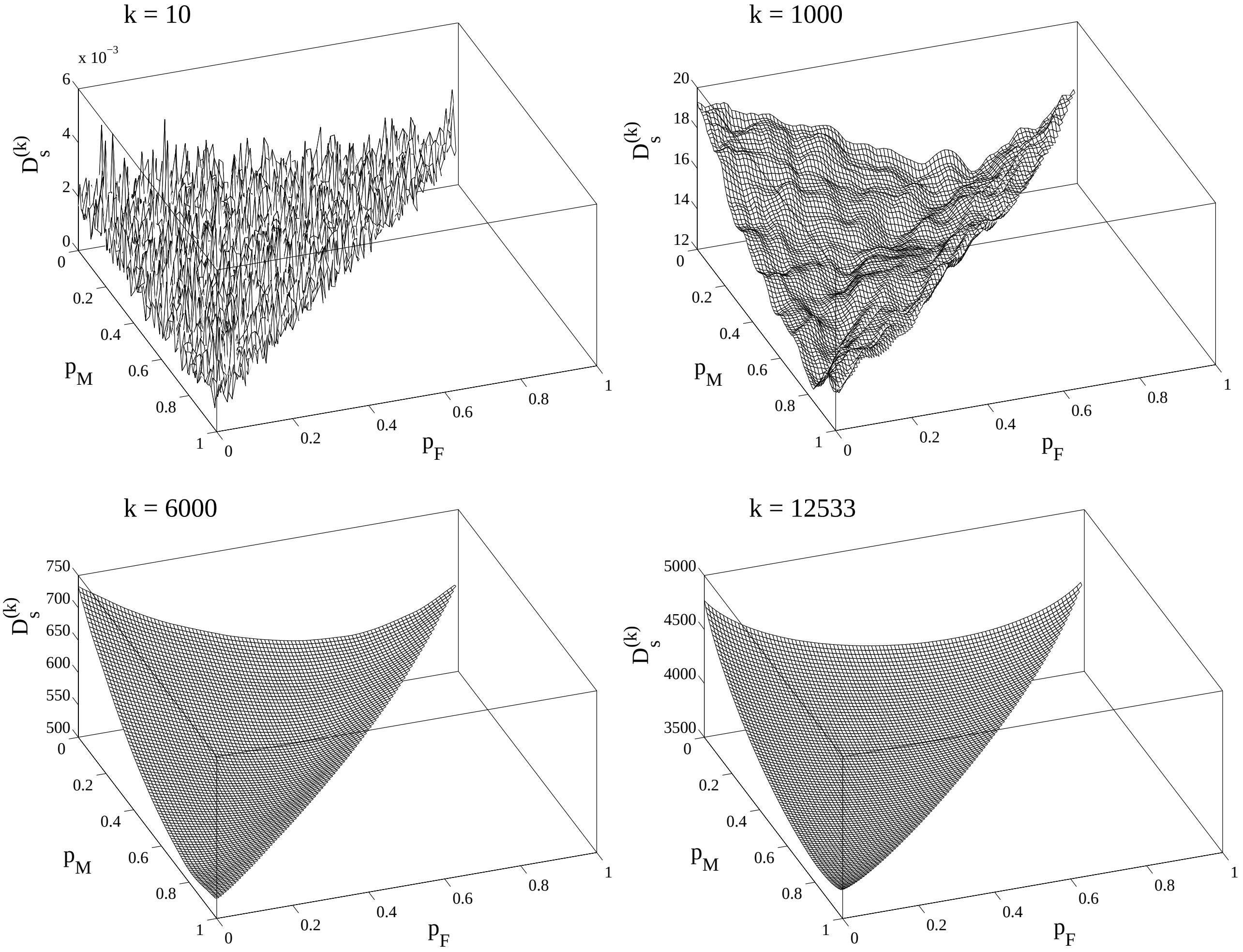

Figure A.3: Objective function $D_s^{(k)}$ for the data of patient 2 as a function of the relative mRNA proportions p_M and p_F. For a small number of included genes k the objective function is very rough and has a multitude of equivalent local minima. The overall flatness of the objective function on a large scale results in approximately equally distributed proportions in the triangle $0 \leq p_M + p_F \leq 1$ if the computations are initialized by equally distributed random starting values. An equal distribution within this triangle results in identical marginal probability density functions (PDFs) for p_M and p_F and thus in equal mean values ($p_M = p_F = 1/3$). As k increases the number of local minima reduces and a global minimum emerges. The macrophage, fibroblast, and non-adherent cell proportions determined for patient 2 read $p_M = 0.82, p_F = 0.09$, and $p_N = 0.09$ ($= 1 - p_M - p_F$), respectively (Table 1 of the main text, MAS-S t), and correspond to the minimum of $D_s^{(k)}$ at $k = 6000$ included genes. For large values of k the location of the global minimum is shifted towards an equal distribution ($p_M = p_F = p_N = 1/3$) and the minimizing proportions at $k = 12533$ read $p_M = 0.65, p_F = 0.23$, and $p_N = 0.11$, respectively (see also Figure 2 of the main text).

The objective function turned out to be very similar for L_1 (least modulus) and L_2 (least squares) regression. Generally, L_1 regression was used in the present study because it was expected to be more robust with respect to y-outliers. For comparison, the results for patient 2 using L_2 regression are shown in Figure A.4

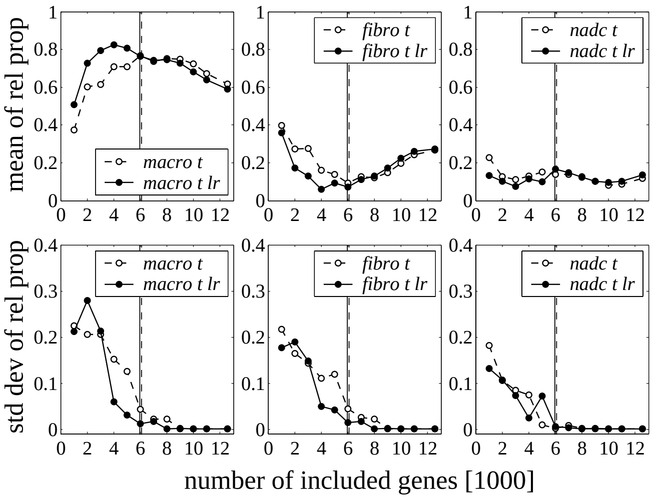

Figure A.4: Mean and standard deviation of the computed relative mRNA proportions for macrophages (*macro*), fibroblasts (*fibro*) and non-adherent cells (*nadc*) for patient 2 as a function of the number k of genes included in the trimmed regression approach. The graphic is analogous to Figure 1 of the main text except that trimmed L_2 (least squares) regression is used instead of L_1 (least modulus) regression. The mRNA proportions are estimated to be $p_M = 0.77/0.76\,(lr)$ for macrophages, $p_F = 0.09/0.07\,(lr)$ for fibroblasts and $p_N = 0.14/0.17\,(lr)$ for non-adherent cells. These values were determined at 6000 included genes.

B Experimental Materials and Methods

Pieces of synovial tissue obtained from 3 patients fulfilling the American Rheumatism Association criteria for rheumatoid arthritis (RA, patients 1-3) [6] and 3 patients with osteoarthritis (OA, patients 4-6) [7], were placed in RNAlater (Ambion Inc., Austin, TX, U.S.A.) for 24 h at room temperature and then frozen immediately at -70°C. The different synovial cell populations were prepared as previously described [8]. Briefly, the tissue samples were minced, digested with trypsin and collagenase P, and the resulting single cell suspension cultured for 7 days. During this primary culture, **non-adherent cells** were removed by medium exchange on day 1. After 7 days, **synovial fibroblasts** were purified (98% purity) by removal of **macrophages** using Dynabeads® M-450 CD14 and the Dynal magnetic particle concentrator®. Non-adherent cells, fibroblasts and Dynabeads-coupled macrophages (>95% purity) were immediately lysed in RLT buffer (Qiagen, Hilden, Germany) and frozen at -70°C. Total RNA was isolated using the RNeasy Kit (Qiagen) according to the supplier's recommendation. RNA probes were labeled according to the supplier's instructions (Affymetrix, Santa Clara, CA, USA). Analysis of gene expression was carried out using HG-U95A (patient 1) and HG-U95Av2 (patient 2-6) chips. Hybridization and washing of the gene chips was performed according to the supplier's instructions (http://www.affymetrix.com). Microarrays were analyzed by laser scanning (Hewlett-Packard Gene Scanner). The synovial tissue chip of patient 1 showed about twice as much noise as the other chips according to the operating figures provided by Affymetrix' MAS 5.0 software. Therefore, the hybridization was repeated twice (using HG-U95Av2 chips), the second repeat having been more stringently washed. Finally, the first repeat was selected for analysis because its operating figures were most compatible with the remaining chips. The purity of the isolated cell fractions was confirmed by a strong down-regulation of specific marker genes for macrophages, fibroblasts, and T-cells (a component of non-adherent cells) in the respective other cell fractions. Two own mixing experiments were performed, in which mRNA preparations of the isolated cell fractions of patient 2 were mixed according to two sets of relative mRNA proportions (that are representative for the range of computed values) and were then hybridized to a HG-U95Av2 chip.

Immunohistochemical analysis was performed on cryostat sections of RA synovial membranes (fixed with acetone for 10 min at 4°C) using the following monoclonal antibodies (mAbs): AS02 (CD90; Dianova GmbH, Hamburg, Germany) and 3-2B12 (prolyl-4-hydroxylase; Merck Biosciences, Schwalbach, Germany; both fibroblast markers); CLB CD14B (CD14; Janssen Biochimica, Beerse, Belgium; monocyte/macrophage marker); UCHT-1 (CD3; Dako, Hamburg, Germany; T-lymphocyte marker); HIB19 (CD19; BD, Heidelberg, Germany; B-lymphocyte marker); AT13/5 (CD38; Dako; plasma cell marker); 4F9 (van Willebrand Factor; Beckman Coulter, Krefeld, Germany; endothelial cell marker). The primary mAbs, diluted in phosphate-buffered saline (PBS)/1% horse serum, were added to the tissue sections for 30 min in a humid chamber at room temperature following pre-incubation with 5% normal horse serum in PBS for 20 min. A peroxidase-coupled rabbit anti-mouse antibody (Dako; 1:30 diluted in PBS/1% horse serum) was then added for 30 min. The peroxidase was revealed under visual control for approx. 5 min using diaminobenzidine (0.5 mg diaminobenzidine in 1 ml PBS containing 30 μl H_2O_2). The slides were washed, counterstained with haematoxylin and covered with Aquatex (Merck). Isotype-matched control mouse mAbs, used at identical concentrations as the primary antibodies, gave no positive results.

For the assessment of the cellular composition, one to three different synovial tissue sections from each patient were analyzed. The cellular composition was separately assessed in the following compartments: lining layer, diffuse infiltrates, lymphoid aggregates, and stroma. The area of the individual compartments was measured in each section using the software AxioVision 4.2 (Carl Zeiss Vision GmbH, Hallbergmoos, Germany) and the relative percentage of cells showing a positive reaction for each marker in each compartment was then visually estimated in 10% steps. In addition, the cellular density of the respective compartment was determined by counting the cell nuclei in two to five 150 μm^2 areas. The percentage of cells positive for each marker in each compartment was then corrected for: i) the relative area of the compartment in the tissue section; and ii) the cell density of the compartment.

C Comparison of MAS-S and MAS-C probe set summaries

The MAS-S probe set summaries were generally preferred to the MAS-C, RMA, and MBEI summaries because they showed a better agreement between the mRNA proportions computed with and without additional stepwise local regression normalization. This is demonstrated by comparing the results obtained from MAS-S and MAS-C for patients 4 and 1 in Figures C.1 and C.2, respectively. The computed relative mRNA proportions are displayed in Table C.1.

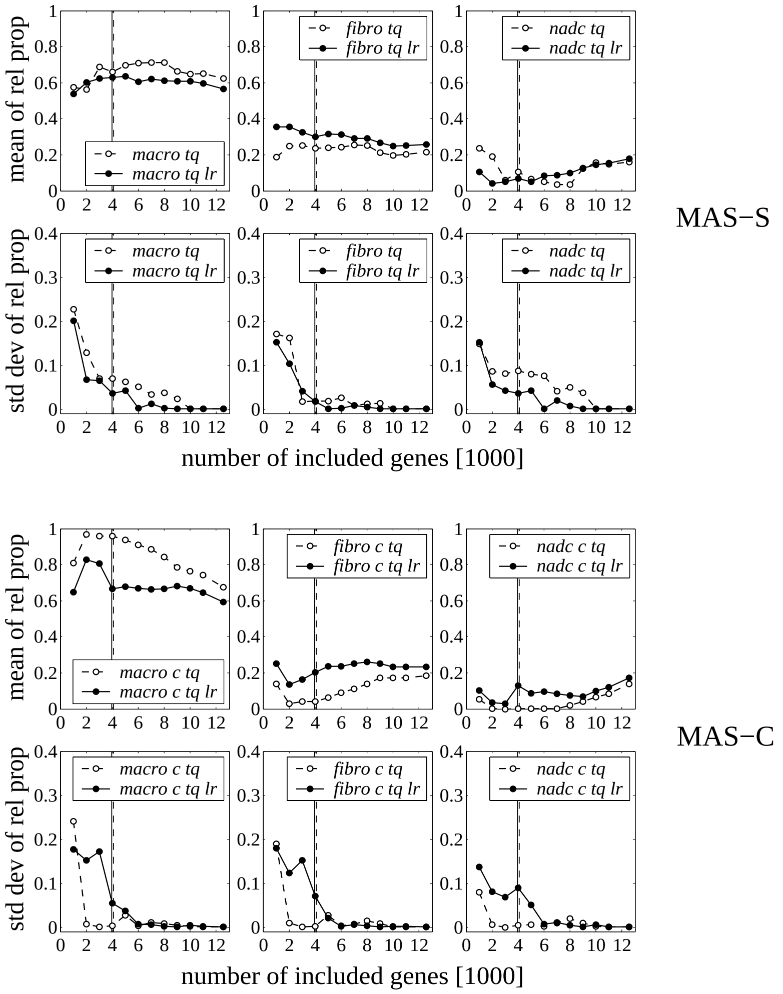

Figure C.1: Mean and standard deviation of the computed relative mRNA proportions for macrophages (*macro*), fibroblasts (*fibro*) and non-adherent cells (*nadc*) for **patient 4** as a function of the number of included genes. MAS-S signal algorithm (upper panel) and MAS-C chip comparison algorithm (c) (lower panel). The measured expression profiles are trimmed mean (t) and quantile (q) normalized. The reconstituted tissue profile is either not further normalized (dashed) or additionally normalized by stepwise local regression (lr) (solid). The differences between the curves without further normalization and additional stepwise local regression normalization (lr) are markedly larger for MAS-C compared to MAS-S. The points chosen for the determination of the relative mRNA proportions are indicated by the dashed and solid (lr) vertical lines.

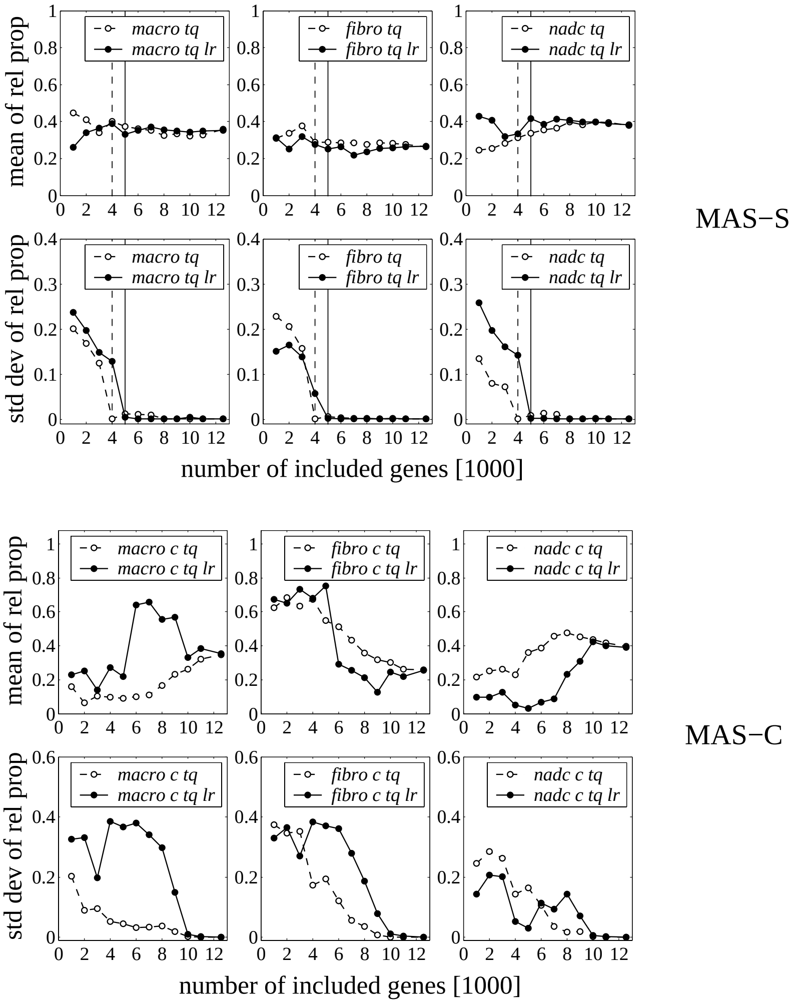

Figure C.2: Mean and standard deviation of the computed relative mRNA proportions for macrophages (*macro*), fibroblasts (*fibro*) and non-adherent cells (*nadc*) for **patient 1** (original tissue chip) as a function of the number of included genes. The measured expression profiles are MAS-S (upper panel) and MAS-C (c) (lower panel) probe set summaries normalized by a symmetric trimmed mean (t) and quantile normalization (q). The reconstituted tissue profile is either not further normalized (dashed) or normalized by stepwise local regression (lr) (solid). Similar results were obtained for the other three normalization methods ($t, t\,clr, t\,c$; for notation see Table 1 of the main text).

chip evaluation method	patient 1' (RA)				patient 2 (RA)				patient 3 (RA)				patient 4 (OA)				patient 5 (OA)				patient 6 (OA)			
			lr				*lr*				*lr*				*lr*				*lr*				*lr*	
	M	*F*	*M*	*F*	*M*	*F*	*M*	*F*	*M*	*F*	*M*	*F*	*M*	*F*	*M*	*F*	*M*	*F*	*M*	*F*	*M*	*F*	*M*	*F*
MAS-S *t*	29	16	29	19	82	9	79	6	64	27	64	29	57	24	57	27	72	21	69	20	86	13	81	19
MAS-S *t clr*	32	18	29	22	90	10	86	5	66	30	64	33	65	25	64	30	74	21	68	25	78	22	74	26
MAS-S *t q*	31	14	24	22	92	8	93	6	69	30	63	34	66	23	63	30	87	07	71	22	71	29	67	33
MAS-S *t c*	31	15	29	17	80	5	80	6	59	31	65	30	71	16	66	21	80	13	80	11	96	2	80	20
MAS-C *t*	—	—	—	—	98	1	91	7	69	22	71	27	63	27	59	27	75	17	75	13	98	1	100	0
MAS-C *t clr*	—	—	—	—	100	0	97	2	70	30	64	34	96	4	72	24	92	1	69	28	98	0	95	5
MAS-C *t q*	—	—	—	—	100	0	97	2	98	1	69	30	96	4	67	20	95	4	72	25	95	5	95	5
MAS-C *t c*	—	—	—	—	98	1	97	3	66	24	71	27	72	22	60	26	85	6	74	14	97	2	100	0

Table C.1: Computed relative mRNA proportions p_M of macrophages and p_F of fibroblasts for patients (1)2-6 calculated for different chip evaluation methods. The proportion of non-adherent cells is $p_N = 1 - p_M - p_F$. Additional stepwise local regression normalization is indicated by *lr*. The proportions are given in percent. Probe set summaries: MAS-S: Microarray Suite 5.0 signal algorithm, MAS-C: Microarray Suite 5.0 chip comparison algorithm, Normalization methods: *t*: trimmed mean, *clr*: cyclic local regression, *q*: quantile normalization, *c*: centralization. MAS-C was not evaluated for the repeated experiments of patient 1 because it was not possible to use HG-U95Av2 chips as baseline experiments for HG-U95A chips in MAS 5.0 and MAS-C was not yet implemented in the most recent version (1.5.8) of the BioConductor affy package.

D Experimental chip quality

The differences in the computed mRNA proportions resulting from different chip evaluation methods, i.e. probe set summaries and chip normalizations, could be caused by varying experimental chip quality. In order to address this hypothesis, the MAD of the computed mRNA proportions (as defined in Table 2 of the main text) was correlated with several relevant chip operating figures provided by the Affymetrix ‘Expression Report’. The operating figures estimate the background and noise level, as well as the degree of mRNA degradation for each chip. After scaling by the ‘Scale Factor (SF)’ of the respective chips, the operating figures were summarized for each patient by taking a weighted average across the four chips per patient.

With a few exceptions, the MAD of the calculated mRNA proportions was positively correlated with the noise and background level (Figure D.1). The correlation coefficients ranged from 0.60 to 0.93. However, due to the small number of patients the results strongly depended on individual patients (inhomogeneity correlation), in particular on patient 1 (original tissue chip). The methodological scatter and the correlation with the operating figures were considerably reduced when only the four different normalization methods for the MAS-S summaries were considered (Figure D.2). The correlations became negative (correlation coefficients down to - 0.89), when the additional stepwise local regression normalization was applied to the differently normalized MAS-S summaries, indicating that local regression can make the computed proportions more homogeneous in the presence of noise.

The correlation between the MAD of the computed relative proportions and mRNA degradation (as quantified by the 3’/5’ expression ratios of GAPDH and β-actin) was generally weak, quite unsystematic and mostly negative. This suggests that not mRNA degradation, but rather experimental noise and background are the main parameters affecting the determinability of the tissue mRNA proportions.

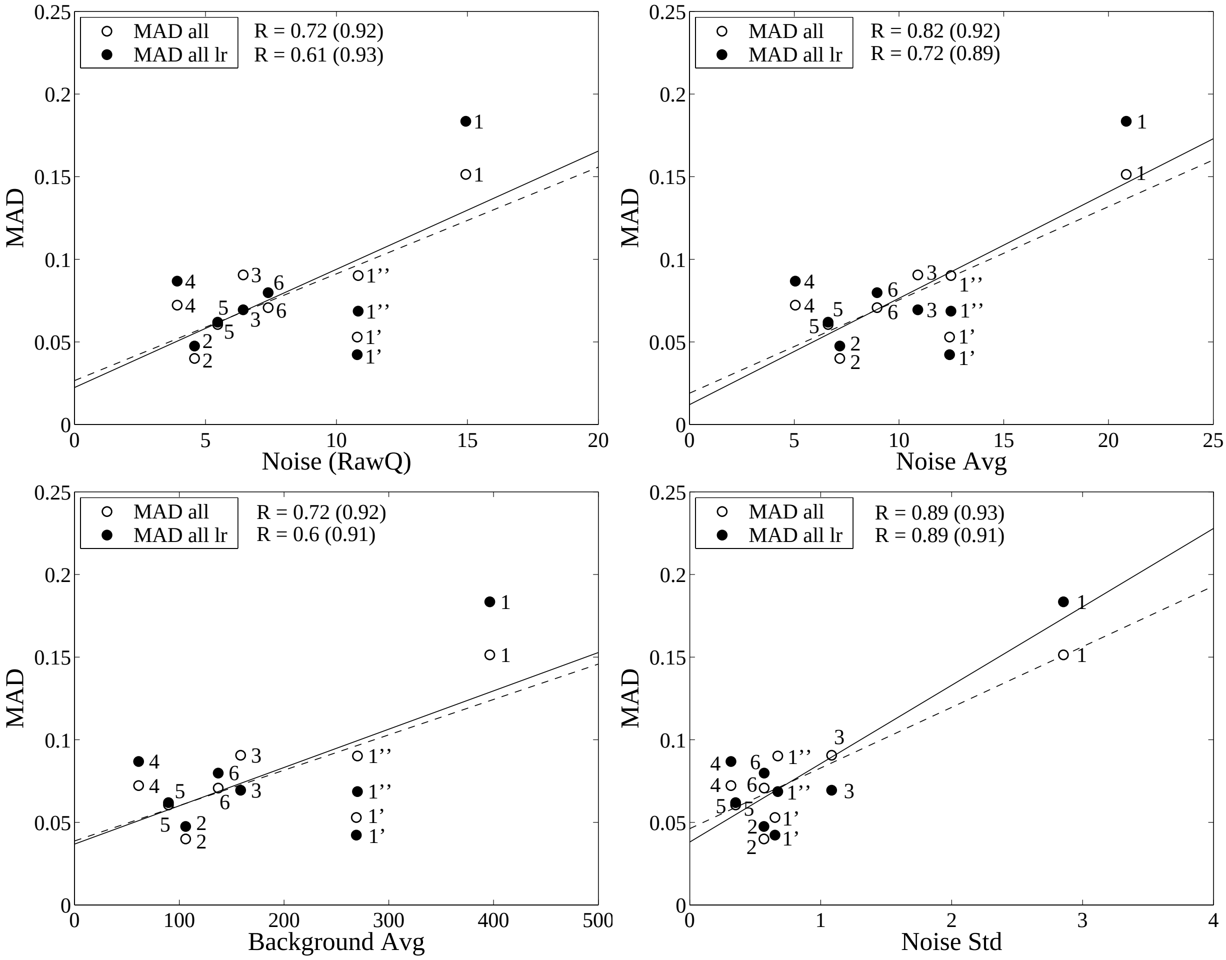

Figure D.1: Pooled mean absolute deviation (MAD) of the computed relative mRNA proportions calculated with respect to all (seven) different chip evaluation methods (MAS-S with four normalization methods, as well as RMA, MBEI and MBEI-GP) versus the weighted average of four background and noise operating figures provided by the ’Expression Report’ of Affymetrix. The point labels indicate the identification number of the patients. The open and closed symbols correspond to whether or not an additional stepwise local regression normalization (lr) was performed. R denotes the Pearson correlation coefficient. The numbers in parentheses give the correlation coefficients if the second and third technical replicates (1’ and 1”) for patient 1 are excluded.

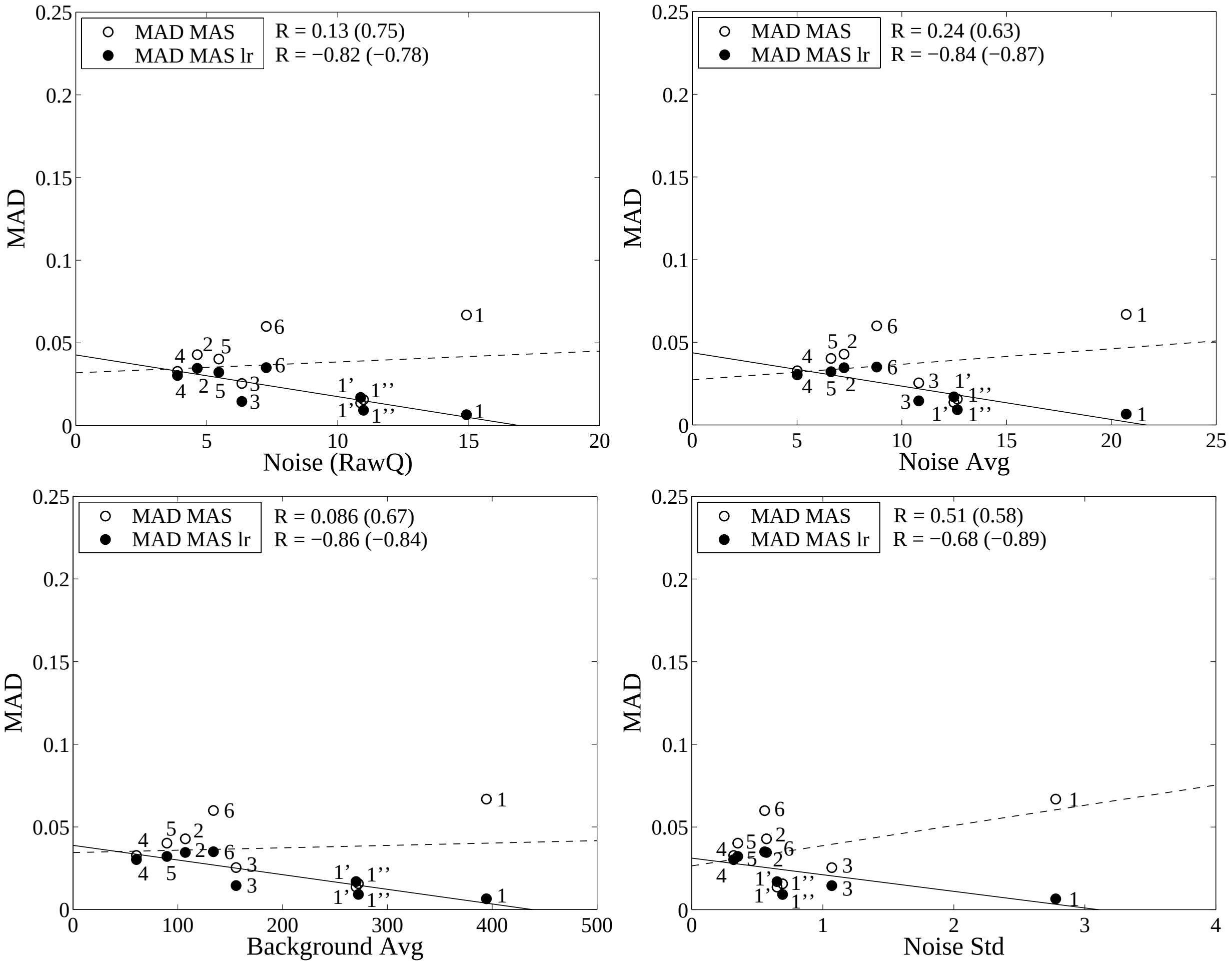

Figure D.2: Pooled mean absolute deviation (MAD) of the computed relative mRNA proportions calculated with respect to the four different normalization methods applied to the MAS-S summaries versus the weighted average of four background and noise operating figures. Notation according to Figure D.1.

E Immunohistochemistry

Protocol for the assessment of the cell type proportions in histological tissue sections

1. Localization of compartments
2. Determination of the respective compartment area (A_{comp})
3. Determination of the cell density in each compartment (ρ_{comp})
   (counting of cell nuclei and averaging over 2-5 pieces with an area of 150 μm^2 each)
4. Computation of the number of cells in each compartment ($N_{comp} = \rho_{comp} \cdot A_{comp}$)
5. Determination of the total number of cells in the tissue section ($N_{total} = \sum N_{comp}$)
6. Assessment of the proportion of marker-positive cells in each compartment (m_{comp})
7. Calculation of the proportion of marker-positive cells in the total section $m_{total} = \left(\sum m_{comp} N_{comp}\right) / N_{total}$

The immunohistological assessment of the cellular composition in synovial tissue sections showed that fibroblasts represented approximately 1/2 of the cells (mean 52% in Table 5 of the main text), followed by macrophages (28%), endothelial cells (9%), T-cells (5%), as well as B-cells and plasma cells (both 2%). Due to the marked heterogeneity among patients, there were no significant differences between tissue samples from RA and OA patients. Non-adherent cells (18%), consisting of T-cells, B-cells, plasma cells, and endothelial cells, showed a large variability among patients (in particular RA patients). T-cells, for example, accounted for approximately 14% of the cells in RA patient 1, but were virtually absent in the other two RA patients. This variability was also observed for B-cells, plasma cells, and endothelial cells, and possibly reflects histological subgroups of RA patients. A higher proportion of endothelial cells in the synovial tissue of RA patients (mean 12%) compared to OA patients (mean 6%) may reflect the high vascularization of the inflamed tissue.

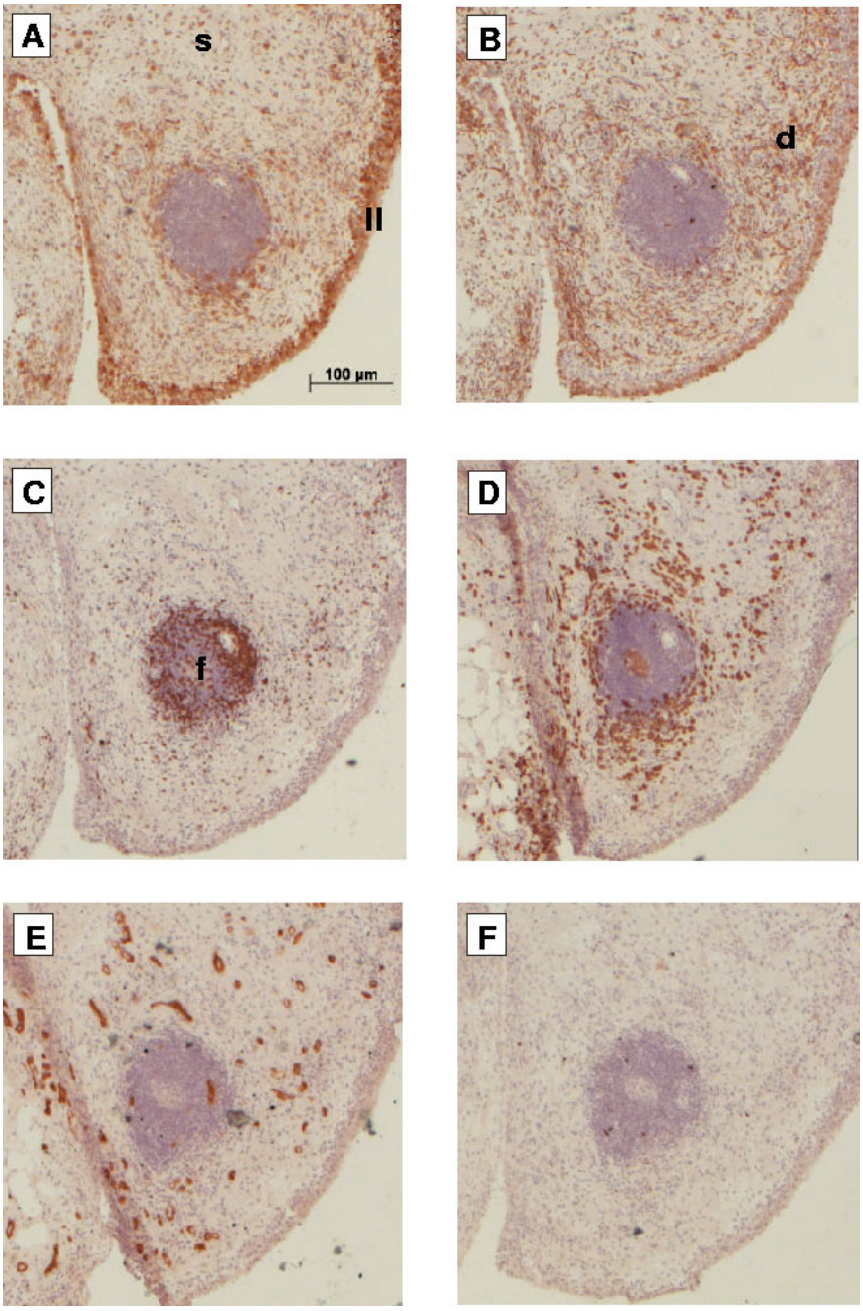

Figure E.1: Immunohistochemical staining of serial sections from synovial tissue (patient 1, RA) showing [A] prolyl-hydroxylase+ fibroblasts in lining layer (ll) and stroma (s), [B] CD14+ macrophages in diffuse infiltrates (d), [C] CD3+ T-cells in a lymphoid follicle (f), [D] CD38+ plasma cells, [E] van Willebrand Factor+ endothelial cells (in each case brown peroxidase staining for positive cells); nuclei were counterstained with haematoxylin (blue staining). [F] shows the negative control (IgG1 isotype antibody) for the respective marker stainings with few peroxidase-positive polymorphonuclear neutrophil granulocytes.

F Cell type-specific marker genes

Stable cell type-specific marker genes allow to determine the mRNA proportions from their expression value in the synovial tissue and the respective isolated cell fraction (e.g., $p_F = s_i/f_i$ for fibroblasts and marker gene i). In the present study, the correspondence between the relative mRNA proportions calculated from Robust Computational Reconstitution and from marker genes were reasonably consistent only for fibroblasts (Table F.1). The results for macrophages and non-adherent cells (using T-cell marker genes) could not be distinguished from random.

gene name	probe set	patient 1'	patient 2	patient 3	patient 4	patient 5	patient 6
lysyl oxidase-like protein gene	36811_at	**<u>16</u>**	34	82	37	102	29
lysyl oxidase (LOX) gene, exon 7	38637_at	6	21	86	**17**	43	**21**
tropomyosin mRNA	36791_g_at	**10**	67	51	9	66	**<u>16</u>**
alpha-1 type XI collagen (COL11A1)	37892_at	**<u>21</u>**	105	555	9	103	121
fibroblast muscle-type tropomyosin	32313_at	**9**	95	**35**	**30**	42	31
elastin mRNA	31621_s_at	**25**	**15**	**<u>27</u>**	3	**<u>22</u>**	**6**
computed by RCR		16	9	27	24	21	14

Table F.1: Relative mRNA proportions for fibroblasts calculated from cell type-specific marker genes. Proportions that deviate less than 10% from the proportions computed using Robust Computational Reconstitution (RCR, bottom line) are typed in bold face. In addition, proportions that deviate less than 5% are underlined.

G Regulated and robustly-expressed genes

Regulated and robustly-expressed genes were selected according to large and small differences, respectively, between their gene expression in the tissue and the respective isolated cell fractions (reconstituted tissue profiles) for all patients. Six different statistical methods (described in Section 2.5 of the main text) were used for this purpose. Each individual method was independently applied for gene ranking. A number of top-ranking genes were defined according to thresholding values (Table G.1) and subsequently screened for pathophysiological relevance. The genes identified as regulated and robustly-expressed, along with their particular identification methods, were finally listed in Tables G.2 and G.3, respectively. Figure G.1 shows the correlation between the six methods and the overlap of the respective 200 top-ranking genes. Figure G.2 presents the relationship between the gene expression strength and the respective statistical selection criteria (p- or λ-values).

method	regulated genes			robustly-expressed genes		
	p-value (λ) threshold	number of genes	method label	p-value (λ) threshold	number of genes	method label
SAM	$1 \cdot 10^{-4}$	191	s	0.980	141	S
t-test1	$1 \cdot 10^{-4}$	148	t_1	0.980	124	T_1
t-test2	$5 \cdot 10^{-5}$	229	t_2	0.980	143	T_2
V&S	60	135	v	0.005	284	V
μ-test	$5 \cdot 10^{-4}$	146	μ	0.980	146	Γ
MAD-test	$5 \cdot 10^{-3}$	137	m	0.994	152	M

Table G.1: Statistical testing methods, thresholding p-values (λ for V&S), number of genes falling into the respective ranges, and method labels used for the selection of regulated and robustly-expressed genes in Tables G.2 and G.3. Methods: SAM: Significance Analysis of Microarrays, t-test1: one-sample paired t-test, t-test2: homoscedastic two-sample t-test, V&S: VERAandSAM, μ-test and MAD-test: μ-test and MAD-test statistics according to Section 2.5 of the main text.

	SAM	t−test1	t−test2	V&S	μ−test	MAD−test
SAM		62<br>177	117<br>187	85<br>16	109<br>175	107<br>9
t−test1	0.928		79<br>178	43<br>13	35<br>161	33<br>4
t−test2	0.977	0.949		50<br>15	46<br>163	44<br>5
V&S	0.753	0.644	0.698		93<br>17	93<br>14
μ−test	0.958	0.844	0.881	0.782		192<br>16
MAD−test	0.501	0.240	0.360	0.629	0.677	

Figure G.1: Lower triangular matrix: Pearson correlation between the p- or λ-values of the individual statistical methods (see Table G.1). The SAM, t-test1, t-test2, and μ-test methods are well correlated. The λ-values of V&S were transformed to the 0-1 range according to $\lambda\text{-transf} = [\lambda' - \min(\lambda')] / [\max(\lambda') - \min(\lambda')]$ using $\lambda' = -\operatorname{arcsinh}(4\,\lambda)$. Upper triangular matrix: number of genes common to the respective top-200 lists of regulated (upper number) and robustly-expressed (lower number) genes obtained from the individual statistical methods (overlap between methods). The average overlap is 79.2 for regulated and 76.7 for robustly-expressed genes. The largest overlap for regulated genes is between μ-test and MAD-test, that for robustly-expressed genes is between SAM and t-test2.

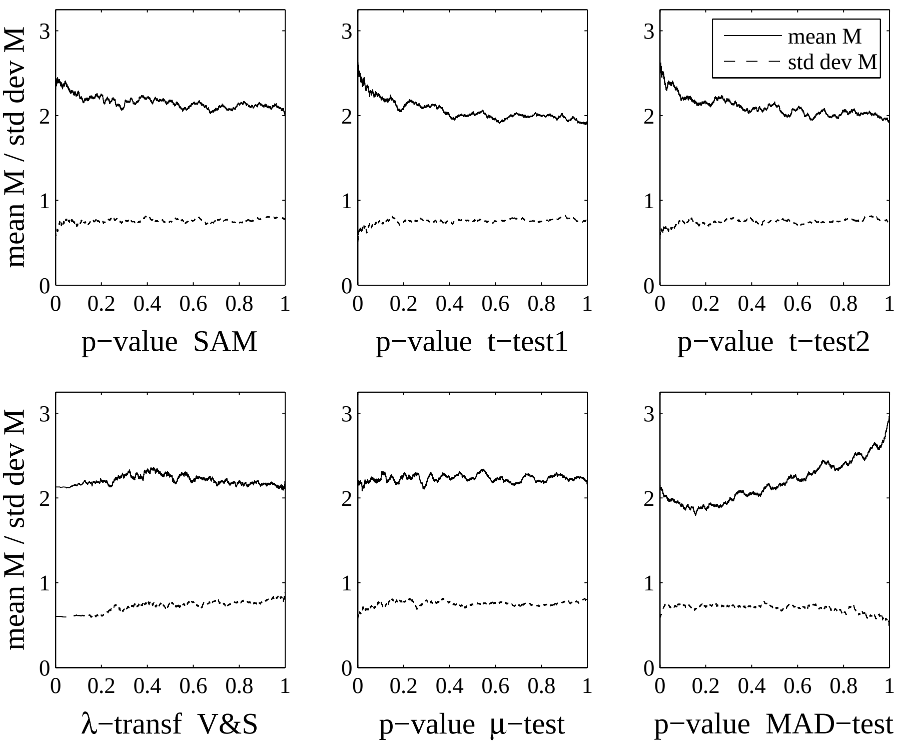

Figure G.2: Moving average (solid) and standard deviation (dashed) of the mean log-transformed gene expression $\mathrm{M} = \frac{1}{2}\left(\log_{10} S + \log_{10} S^*\right)$ of the measured (S) and reconstituted (S^*) tissue as a function of the p-value (transformed λ for V&S, see caption of Figure G.1) of the six different statistical methods used. SAM, t-test1, and t-test2 have a tendency to select highly-expressed regulated genes (low p-value range) and weakly-expressed robustly-expressed genes (high p-value range). The selection by V&S and μ-test appears to be almost unbiased with respect to M. The MAD-test has a clear preference for highly-expressed genes at the upper p-value end (robustly-expressed genes). This may reflect the smaller differences $\mathrm{A} = \log_{10} S - \log_{10} S^*$ observed for highly-expressed genes (large M) in so-called M-A-plots (see also Figure H.3). Moving averages and standard deviations were calculated using a symmetric 200-genes window.

gene name	symbol	probe set	methods	< fold >	< S >	< S^* >	P	P^*
**Immune response**								
chemokine (C-C motif) ligand 14 / ligand 15	CCL14 / 15	33790_at	s,t_1,t_2,v,μ,m	38.3	1238.3	32.3	6	0
chemokine (C-C motif) receptor 2	CCR2	39936_at	s,v,μ,m	17.7	168.0	9.5	3	0
chemokine (C-X3-C motif) receptor 1	CX3CR1	40646_at	s,t_1,t_2,v,μ,m	16.7	372.6	22.4	6	2
CD52 antigen (CAMPATH-1 antigen)	CD52	34210_at	μ,m	12.6	548.2	43.4	6	3
chemokine (C-C motif) ligand 19	CCL19	36067_at	v,μ,m	10.7	228.3	21.2	2	2
Fc fragment of IgG, receptor (CD16a)	FCGR3A	37200_at	v,μ,m	8.9	2562.2	287.6	6	9
Fc fragment of IgG, receptor (CD16b)	FCGR3B	31499_s_at	μ,m	7.5	732.6	97.6	6	4
chemokine (C-X-C motif) ligand 12	CXCL12	32666_at	s,t_2,v	5.9	4391.0	742.6	6	18
chemokine (C-X-C motif) ligand 12	CXCL12	33834_at	s,t_2	4.5	4137.2	920.4	6	17
interleukin 11 receptor, alpha	IL11RA	496_s_at	t_2	3.3	639.8	192.5	6	17
interleukin-1 receptor-assoc. kinase 1 binding protein 1	IRAK1BP1	38357_at	t_1	1.7	1413.4	810.6	6	18
interleukin 13 receptor, alpha 1	IL13RA1	359_at	t_1,t_2	-2.4	464.6	1114.7	6	18
chemokine (C-C motif) ligand 3	CCL3	36103_at	s,μ,m	-7.8	380.1	2979.5	6	17
colony stimulating factor 3 (granulocyte)	CSF3	1334_s_at	s,μ,m	-9.4	50.0	469.9	0	6
suppressor of cytokine signaling 3	SOCS3	40968_at	s,μ,m	-9.6	31.1	299.3	0	13
interleukin 1, beta	IL1B	39402_at	μ,m	-9.7	185.0	1803.3	2	12
interleukin 1, beta	IL1B	1520_s_at	s,μ,m	-10.5	188.9	1987.3	1	12
interleukin 1 receptor antagonist	IL1RN	37603_at	μ,m	-11.6	146.7	1696.1	3	14
interleukin 24	IL24	41849_r_at	s,μ,m	-17.2	3.4	58.2	0	11
chemokine (C-X-C motif) ligand 2	CXCL2	37187_at	s,μ,m	-21.8	188.4	4110.0	6	18
chemokine (C-X-C motif) ligand 1 (alpha)	CXCL1	408_at	s,μ,m	-23.8	269.4	6414.9	5	18
chemokine (C-X-C motif) ligand 5	CXCL5	35025_at	s,t_2,v,μ,m	-24.2	58.3	1409.9	4	18
chemokine (C-X-C motif) ligand 3	CXCL3	34022_at	s,t_2,μ,m	-29.8	102.9	3068.4	2	18
interleukin 6 (interferon, beta 2)	IL6	38299_at	s,μ,m	-30.6	218.3	6675.8	5	18
chemokine (C-X-C motif) ligand 6	CXCL6	35410_at	s,t_1,v,μ,m	-40.0	61.2	2448.0	4	18
interleukin 11	IL11	35464_at	s,t_1,t_2,v,μ,m	-49.5	3.4	167.9	0	14
interleukin 24	IL24	41848_f_at	s,t_1,t_2,v,μ,m	-50.5	4.7	236.9	0	11
interleukin 8	IL8	35372_r_at	s,t_2,μ,m	-51.9	64.5	3348.1	4	18
chemokine (C-C motif) ligand 20	CCL20	40385_at	s,t_1,t_2,v,μ,m	-73.1	24.4	1782.2	2	18
interleukin 8	IL8	1369_s_at	s,μ,m	-76.4	80.9	6186.1	1	18
**Transcription**								
runt-related transcription factor 1 (cyclin D-related)	RUNX1T1	35640_at	s,t_1,t_2,v,μ,m	12.7	80.7	6.4	4	0
prostaglandin E receptor 3 (subtype EP3)	PTGER3	39616_at	v	5.5	158.7	28.9	4	5
runt-related transcription factor 1 (cyclin D-related)	RUNX1T1	35638_at	s,t_2	4.1	1648.9	404.0	6	14
**Extracellular matrix**								
matrilin 2	MATN2	32239_at	s,v,μ,m	11.4	131.5	11.5	6	1
collagen, type XXI, alpha 1	COL21A1	35237_at	s,t_2	3.5	265.4	76.1	4	3
O-linked N-acetylglucosamine (GlcNAc) transferase	OGT	39507_at	t_1	2.6	1464.9	555.7	6	18
tumor necrosis factor receptor 11b (osteoprotegerin)	TNFRSF11B	37611_at	s,t_2	-4.6	61.0	278.3	6	18

Table G.2: Regulated genes associated with immune response, transcription, and extracellular matrix. Gene name and probe set ID according to Affymetrix. Gene symbol according to HUGO Gene Nomenclature Committee (HGNC). Statistical selection methods abbreviated according to Table G.1. Mean fold change across patients $< \text{fold} >= \text{sign}(\text{r}) \exp(|\text{r}|)$, with $\text{r} = \text{mean}(\log(S/S^*))$, S measured tissue gene expression, and S^* reconstituted tissue gene expression. Mean expression $< S >= \exp(\text{mean}(\log S))$, $< S^* >= \exp(\text{mean}(\log S^*))$. P: number of present calls for the 6 synovial tissue chips, P^*: number of present calls for the 18 isolated fraction chips. Data: trimmed mean normalized MAS-S probe set summaries. Number of patients: $n = 6$. Gene ranking was exclusively performed using the statistical methods. The values of $< \text{fold} >, < S >, < S^* >, P$, and P^* are only given as additional information.

gene name	symbol	probe set	methods	< fold >	< S >	< S^* >	P	P*
**Growth factor/signaling**								
insulin-like growth factor 1 (somatomedin C)	IGF1	1501_at	s,μ,m	8.3	1400.3	168.2	6	17
insulin-like growth factor 1 (somatomedin C)	IGF1	38737_at	s,v,μ,m	7.6	383.3	50.3	6	11
thrombospondin 3	THBS3	32670_at	v	6.4	772.1	121.0	5	3
thrombospondin 4	THBS4	103_at	v	5.3	4282.7	801.4	6	18
transforming growth factor, beta receptor III	TGFBR3	1897_at	s,t_2	4.7	1781.4	381.7	6	18
bone morphogenetic protein 4	BMP4	40333_at	t_1,t_2	2.6	491.5	191.9	6	16
insulin-like growth factor 1 receptor	IGF1R	34718_at	t_1	-2.8	71.1	197.3	6	18
transforming growth factor, beta receptor I	TGFBR1	1957_s_at	s,t_2	-5.3	35.1	186.9	0	17
fibroblast growth factor 2 (basic)	FGF2	1828_s_at	s	-6.4	103.2	657.3	5	18
transforming growth factor, beta receptor I	TGFBR1	32903_at	s,t_2	-6.6	27.7	182.0	2	18
bone morphogenetic protein 2	BMP2	1113_at	s	-7.1	82.4	581.4	2	18
insulin receptor substrate 1	IRS1	850_r_at	μ,m	-8.6	4.1	35.2	0	3
thrombospondin 1	THBS1	115_at	μ,m	-8.7	79.6	693.7	1	17
inhibin, beta A (activin A, activin AB alpha polyp.)	INHBA	40357_at	s,t_1,t_2,μ,m	-9.7	37.1	358.4	2	15
phosphodiesterase 3A, cGMP-inhibited	PDE3A	34650_at	s,t_2,μ,m	-13.2	12.9	169.2	0	8
serpin peptidase inhibitor, clade E (nexin)	SERPINE1	672_at	μ,m	-15.7	113.8	1791.3	1	16
**Proteolysis**								
serpin peptidase inhibitor, clade F	SERPINF1	40856_at	s,t_1,t_2	3.8	7232.4	1919.1	6	18
serpin peptidase inhibitor, clade G (C1 inhibitor)	SERPING1	39775_at	t_2	2.4	11343.3	4655.1	6	18
matrix metallopeptidase 13 (collagenase 3)	MMP13	39632_at	m	-7.1	10.8	77.1	1	15
matrix metallopeptidase 3 (stromelysin 1)	MMP3	437_at	μ,m	-8.5	963.4	8145.7	5	18
serpin peptidase inhibitor, clade E (nexin)	SERPINE2	41246_at	s,t_1,t_2,μ,m	-8.9	734.6	6571.8	6	18
matrix metallopeptidase 1 (interstitial collagenase)	MMP1	38428_at	μ,m,V	-10.8	729.3	7845.8	5	18
matrix metallopeptidase 9 (type IV collagenase)	MMP9	31859_at	s,μ,m	-10.9	503.4	5502.2	6	17
serpin peptidase inhibitor, clade B (ovalbumin)	SERPINB2	37185_at	s,t_2,v,μ,m	-22.0	54.6	1201.3	0	16
matrix metallopeptidase 10 (stromelysin 2)	MMP10	1006_at	s,μ,m	-32.1	7.8	252.1	0	12

Table G.2: (Continued) Regulated genes associated with growth factor/signaling and proteolysis.

Comment: Matrix metallopeptidase 1 (MMP1, Proteolysis) is identified as regulated by μ-test and MAD-test in Table G.2, and as robustly-expressed by V&S in Table G.3. Mean fold change (-10.8) and mean expression values (729.3 and 7845.8, respectively) suggest MMP1 to be strongly regulated. The discrepancy with respect to V&S can be attributed to an outlier value for the synovial tissue gene expression of patient 1. This value is approximately 2 times higher than that of the reconstitution, whereas it is 2 to 356 times lower for the other patients. More specifically, the expression values of patients 1 / . . . / 6 are given by $S = 15424.8\,/\,20.6\,/\,25.6\,/\,1301.1\,/\,4515.7\,/\,3139.2$ and $S^* = 7602.6\,/\,7336.7\,/\,7619.9\,/\,7681.2\,/\,9338.1\,/\,7651.0$, respectively. These values result in a mean $\log_2$-ratio of -3.43 with a standard deviation of 3.98. It is probably this large standard deviation that causes V&S to assign the error model corrected mean values 9070.4 and 9022.3 to S and S^*, respectively.

gene name	symbol	probe set	methods	$<$ fold $>$	$< S >$	$< S^* >$	P	P*
**Immune response**								
tumor necrosis factor (TNF superfam., member 2)	TNF	259_s_at	M	1.05	305.6	292.1	2	5
immunoglobulin heavy constant mu	IGHG3	31564_at	V	1.02	117.0	114.1	0	0
interleukin 17 (cytotox. serine esterase 8)	IL17	1359_at	V	1.01	23.3	23.0	0	0
interferon, alpha 16	IFNA16	1075_f_at	T_2	1.01	26.4	26.1	0	0
interleukin 7	IL7	1159_at	S,T_1,T_2	1.01	31.0	30.7	1	5
colony stimulating factor 3 receptor (granulocyte)	CSF3R	34223_at	S,T_1,T_2,Γ	1.01	205.4	204.3	5	5
interleukin 15	IL15	1036_at	Γ	1.01	75.0	74.6	6	17
Fc fragment of IgG, low aff. IIa, receptor (CD32)	FCGR2A	37688_f_at	S,T_1,T_2,Γ	1.00	356.3	354.9	6	14
macrophage scavenger receptor 1	MSR1	39982_r_at	S,T_1,T_2,Γ	1.00	112.8	112.5	5	8
interferon, alpha 5	IFNA5	32415_at	S,T_1,T_2,Γ	1.00	12.1	12.1	0	1
tumor necrosis factor, alpha-induced protein 8	TNFAIP8	33243_at	M	-1.03	1091.3	1123.1	6	18
interleukin 8 receptor, beta	IL8RB	1032_at	M	-1.06	286.1	304.7	0	0
interferon, alpha 5	IFNA5	1540_f_at	V	-1.46	28.7	42.0	0	0
chemokine (C-X-C motif) receptor 4	CXCR4	649_s_at	V	-2.29	1499.1	3430.7	6	17
**Transcription**								
wingless-type MMTV integr. site fam., member 1	WNT1	1853_at	V,M	1.04	8.0	7.7	0	0
runt-related transcription factor 1 (aml1 oncogene)	RUNX1	31443_at	S,T_1,T_2,V,Γ	1.00	89.0	88.7	4	16
nuclear factor related to kappaB binding protein	NFRKB	39138_g_at	Γ	-1.01	166.4	167.3	4	10
**Extracellular matrix**								
carbohydrate (chondroitin 6) sulfotransferase 3	CHST3	32094_at	V	1.09	631.3	579.1	6	17
procollagen-proline, 2-oxoglutarate 4-dioxygenase	P4HB	36666_at	V	1.04	7617.9	7355.5	6	18
collagen, type I, alpha 2	COL1A2	32308_r_at	S,T_1,T_2	1.01	23.8	23.6	2	4
hyaluronoglucosaminidase 3	HYAL3	35540_at	S,T_1,T_2,Γ	-1.00	19.9	19.9	0	0
N-deacetylase/N-sulfotransferase (heparan gluco.) 2	NDST2	37255_at	S,T_1,T_2,Γ	-1.01	240.5	241.8	4	9
collagen, type XI, alpha 2	COL11A2	1026_s_at	V	-1.18	117.1	137.7	0	0
lysyl oxidase	LOX	38637_at	V	-1.43	1031.5	1476.1	6	18
glucosaminyl (N-acetyl) transferase 2	GCNT2	35033_at	V	-1.46	7.7	11.2	0	0
**Growth factor/signaling**								
phosphodiesterase 5A, cGMP-specific	PDE5A	34953_i_at	V	1.02	15.0	14.7	0	0
phosphoinositide-3-kinase, catalytic, gamma polypept.	PIK3CG	36287_at	T_2	1.01	85.9	85.2	5	9
Insulin receptor substrate 2	IRS2	40181_f_at	S,T_1,T_2,Γ	-1.00	15.5	15.6	0	0
cell division cycle 25C	CDC25C	1584_at	S,T_1,T_2,Γ	-1.00	37.8	38.0	0	4
v-ral simian leukemia viral oncogene homolog B	RALB	32776_at	S,V,Γ	-1.00	725.1	728.1	6	18
platelet-derived growth factor receptor, alpha poly.	PDGFRA	36157_at	T_2	-1.01	5.2	5.2	0	0
SMAD, mothers against DPP homolog 9 (Drosophila)	SMAD9	32986_s_at	V	-1.03	68.6	70.4	0	0
fibroblast growth factor 13	FGF13	36232_at	V	-1.17	11.8	13.8	0	0
vascular endothelial growth factor B	VEGFB	1926_at	V	-1.26	447.9	564.8	4	13
**Proteolysis**								
elastase 2B	ELA2B	31478_at	V	1.14	177.1	155.2	0	0
matrix metallopeptidase 24 (membrane-inserted)	MMP24	32924_at	V	1.02	45.5	44.5	0	0
matrix metallopeptidase 20 (enamelysin)	MMP20	31340_at	T_1,T_2	1.01	12.8	12.7	0	0
zinc metallopeptidase (STE24 homolog, yeast)	ZMPSTE24	33912_at	S,T_1,T_2,Γ	1.00	600.9	599.7	6	18
serpin peptidase inhibitor, clade B, member 5	SERPINB5	862_at	S,T_1,T_2,Γ	-1.00	12.5	12.5	0	0
calpain 1, (mu/I) large subunit	CAPN1	33908_at	S,Γ,M	-1.00	1427.6	1431.1	6	16
matrix metallopeptidase 16 (membrane-inserted)	MMP16	40759_at	S,T_1,T_2,Γ	-1.00	11.2	11.3	0	0
cathepsin O	CTSO	36914_at	T_2	-1.01	20.9	21.1	0	0
caspase 6, apoptosis-related cysteine peptidase	CASP6	35662_at	T_2	-1.01	70.1	71.1	4	12
serpin peptidase inhibitor, clade C, member 1	SERPINC1	37175_at	V	-1.03	6.5	6.7	0	0
matrix metallopeptidase 7 (matrilysin, uterine)	MMP7	668_s_at	V	-1.05	21.1	22.2	1	1
caspase 4, apoptosis-related cysteine peptidase	CASP4	195_s_at	V	-1.07	1060.8	1137.8	6	18
cathepsin L2	CTSL2	40717_at	V	-1.19	26.7	31.7	0	0
matrix metallopeptidase 1 (interstitial collagenase)	MMP1	38428_at	V,μ,m	-10.76	729.3	7845.8	5	18

Table G.3: Robustly-expressed genes associated with immune response, transcription, extracellular matrix, growth factor/signaling, and proteolysis. Notation according to Table G.2.

H Probabilistic model for cell type-specific gene expression

The gene expression values of each individual cell type in the tissue can be estimated from the gene expression values of the isolated cell fractions and the whole tissue based on a suitable, fully specified probabilistic model, i.e. a model in which all distributional parameters are settled. In this section a multivariate normal distribution model is assumed for this purpose. Clearly, the cell type-specific mean expression values in the tissue as well as the respective correlation coefficients between tissue and isolated cell fractions cannot be estimated directly because the cell type-specific gene expression in the tissue is not available. However, these distributional parameters (mean and correlation coefficients) could possibly be approximated as an universal function of expression strength. This general approach of substituting statistical parameters using functions of expression strength can be tested for the standard deviations because they can be calculated in two alternative ways: either data-based using the expression values measured for the isolated cell fractions or by substitution, using a function of expression strength. Although the results of both methods were largely consistent, a number of contradictory results were observed (Table H.1). Consequently, the present substitution approach cannot fully compensate for the lack of experimental microdissection data and thus the cell type-specific gene expression values in the tissue cannot reliably be estimated.

The probabilistic model assumes the log-transformed expression of each gene in each cell type to be a normally distributed random variable (subsequently denoted by lower case letters, omitting the gene index i introduced in Equation (3) of the main text, i.e. $\bar{m} = \log \bar{M}$, $m = \log M$, etc.). The joint multivariate Gaussian probability density function (PDF) of the random vector $\boldsymbol{x} = (\bar{m}, \bar{f}, \bar{n}, m, f, n)^\top$ containing the log-transformed expression values of the three different cell types in the tissue $(\bar{m}, \bar{f}, \bar{n})$ and the isolated cell fractions (m, f, n) reads

$$f(\boldsymbol{x} \,|\, \boldsymbol{\mu}, \Sigma) = \frac{1}{\sqrt{\det(2\,\pi\,\Sigma)}} \exp\left(-\frac{1}{2}\,(\boldsymbol{x}-\boldsymbol{\mu})^\top\, \boldsymbol{\Sigma}^{-1} (\boldsymbol{x}-\boldsymbol{\mu}) \right) \,, \tag{H.1}$$

with $\boldsymbol{\mu}$ denoting the vector of mean values and $\boldsymbol{\Sigma}$ being the covariance matrix: $\Sigma_{\alpha\beta} = r_{\alpha\beta}\,\sigma_\alpha\,\sigma_\beta$ with $r_{\alpha\beta}$ being the correlation coefficient between α and β, and σ_α and σ_β representing the respective standard deviations. In order to ensure the computed tissue expression $\bar{s}(\boldsymbol{x}) = \log\left(p_M\,\exp\bar{m} + p_F\,\exp\bar{f} + p_N\,\exp\bar{n}\right)$ to be close to the measured value s, the PDF in (H.1) was regularized by multiplication with the Gaussian

$$g(\boldsymbol{x} \,|\, s, \hat{\sigma}_s) = \frac{1}{\sqrt{2\,\pi}\,\hat{\sigma}_s}\, \exp\left(-\frac{1}{2\,\hat{\sigma}_s^2}\left(\bar{s}(\boldsymbol{x}) - s\right)^2 \right) \quad . \tag{H.2}$$

Analytical expressions for the expectation values and standard deviations of the cell type-specific changes in gene expression could be obtained by linearizing $\bar{s}(\boldsymbol{x})$ with respect to the differences $\delta_\alpha = \bar{\alpha} - \alpha$ for $\alpha = m, f, n$ (i.e. substituting $\bar{m} = m + \delta_m$ and expanding with respect to δ_m, etc.). Assuming, for notational convenience, equal mean values in tissue and isolated cell fractions, equality of means and expression values among the isolated cell fractions, and considering the cell type-specific correlation coefficients only (i.e. $r_{\alpha\beta} = 0$ except for $\alpha = \beta$ and $\alpha = \bar{\beta}$), the expectation values $\mathcal{E}[\delta_\alpha]$ and standard deviations $\mathcal{S}[\delta_\alpha]$ of the expression differences read

$$\begin{aligned} \mathcal{E}[\delta_\alpha] &= \frac{\delta_s\; w_\alpha}{\boldsymbol{p}\cdot\boldsymbol{w} + \hat{\sigma}_s^2} \quad , \quad w_\alpha \;=\; p_\alpha\,\left(1 - r_{\bar{\alpha}\alpha}^2\right)\,\sigma_\alpha^2 \quad , \\ \mathcal{S}[\delta_\alpha] &= \sigma_\alpha\,\sqrt{(1 - r_{\bar{\alpha}\alpha})\left(1 - \frac{p_\alpha\; w_\alpha}{\boldsymbol{p}\cdot\boldsymbol{w} + \hat{\sigma}_s^2}\right)} \quad , \end{aligned} \tag{H.3}$$

in which $\delta_s = s - s^*$ denotes the observed difference in gene expression, $\boldsymbol{p}\cdot\boldsymbol{w} = \sum_\alpha p_\alpha\,w_\alpha$ is the scalar product between $\boldsymbol{p}$ and $\boldsymbol{w}$, and $p_m = p_M\,(M/S^*)$, etc. The condition $\bar{s}(\boldsymbol{x}) = s$ is exactly met for $\hat{\sigma}_s = 0$ implying $\delta_s = \sum_\alpha p_\alpha\,\mathcal{E}[\delta_\alpha]$. Clearly, the numerator of $\mathcal{E}[\delta_\alpha]$ equally depends on the fraction p_α, one minus the squared correlation coefficient $r_{\bar{\alpha}\alpha}^2$, and the variance σ_α^2, i.e. the correlation coefficients $r_{\bar{\alpha}\alpha}$ need to be known or estimated.

For a better identification of simultaneous changes in groups of patients the above approach was generalized to multiple patients having identical changes δ_α in gene expression. Assuming the patients to be statistically independent the total PDF is the product of the regularized PDFs for each patient introduced in Equations (H.1) and (H.2)

$$h(\delta_m, \delta_f, \delta_n \,|\, \Omega_P) \;=\; \prod_{p=1}^{P}\, g\left(\boldsymbol{x}_p | s_p, \hat{\sigma}_{sp}\right)\; f\left(\boldsymbol{x}_p | \boldsymbol{\mu}_p, \Sigma_p\right) \quad . \tag{H.4}$$

In Equation (H.4) the cell type-specific gene expression values in the tissue were framed as $\bar{\alpha}_p = \alpha_p + \delta_\alpha$ for each patient and $\alpha = m, f, n$. Also, Ω_P summarizes the distributional parameters for all P patients. The standard deviations $\hat{\sigma}_{sp}$ of the regularizing Gaussians were obtained by approximating $\sigma_s = \sigma_s(s)$ as a function of the mean expression

level s using linear regression (Figure H.1) and multiplying the function values $\sigma_s(s_p)$ by a scaling factor, presently 0.1. Similarly, the cell type-specific correlation coefficients $r_{\bar{\alpha}\alpha p}$ were estimated by $r_{ss^*}(\alpha_p)$, i.e. by generalizing the dependence of the correlation coefficient r_{ss^*} between the measured and reconstituted tissue profiles (across patients) on the expression level to the individual cell types (Figure H.2).

For assessing the reliability of the estimation method for $r_{\bar{\alpha}\alpha p}$ the cell type-specific standard deviations $\sigma_{\alpha p}$ were determined in two alternative ways. On the one hand, they were directly calculated from the respective isolated cell fraction (across patients, thus being equal for all patients). On the other hand, they were determined using expression-level-based regression, analogous to the estimation of $r_{\bar{\alpha}\alpha p}$ described above (cf. Figure H.1).

Weakly-expressed genes show a tendency to be upregulated (and vice versa) (Figure H.3). This was incorporated into the model by assuming the differences between the mean values in the tissue and the respective isolated cell fractions to depend on the expression level (again via linear regression). However, the results obtained were very similar to the results reported in Table H.1 for equal means, indicating that this trend is too week to have a distinct effect on the results.

Table H.1 shows the results for some arbitrarily selected genes. The cell type-specific assignment of the changes in tissue gene expression δ_s sometimes was contrary between the two alternative ways of determining the standard deviations $\sigma_{\alpha p}$ described above. Thus, in view of Figure H.2, which clearly shows the weak correlation between expression level and correlation coefficient, a reliable cell type-specific assignment of the changes in gene expression (tissue versus isolated cell fractions) appears to be impossible given the present data.

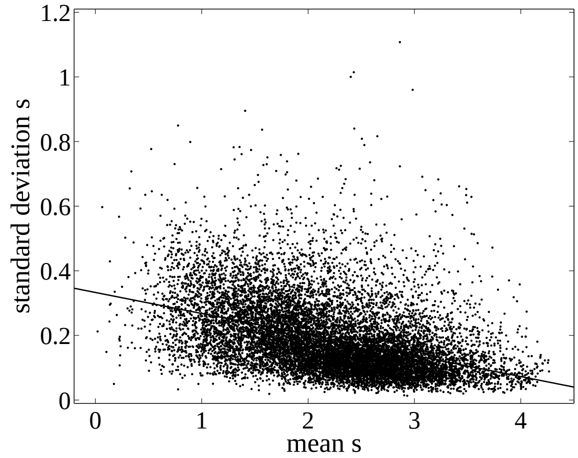

Figure H.1: Standard deviation σ_s of the gene expression $s = \log_{10} S$ in the synovial tissue as a function of the mean of s (both calculated across patients 1-6). Regression line: offset = 0.33, inclination = - 0.07. Pearson correlation coefficient = 0.43.

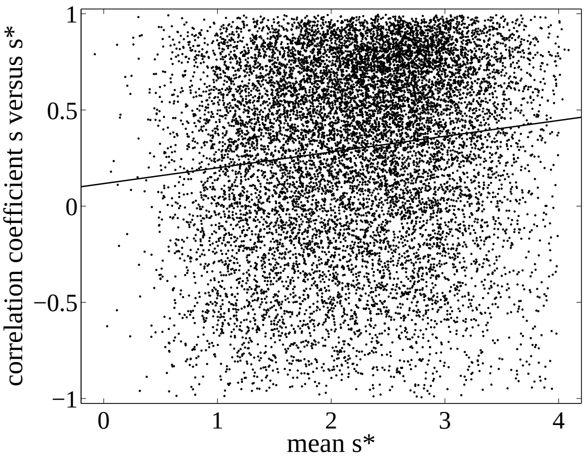

Figure H.2: Correlation coefficient r_{ss^*} between the gene expression $s = \log_{10} S$ and $s^* = \log_{10} S^*$ in the measured and reconstituted tissue, respectively, as a function of the mean of s^* (both calculated across patients 1-6). Regression line: offset = 0.12, inclination = 0.08. Pearson correlation coefficient = 0.13.

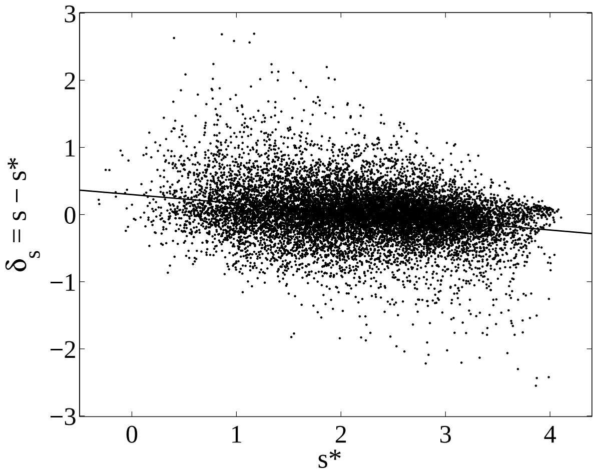

Figure H.3: Difference $\delta_s = s - s^*$ between the gene expression $s = \log_{10} S$ and $s^* = \log_{10} S^*$ in the measured and reconstituted tissue, respectively, as a function of s^* for patient 2. Regression line: offset = 0.30, inclination = - 0.13. Pearson correlation coefficient = 0.26. Weakly-expressed genes tend to be upregulated and vice versa.

standard deviations					expression level-based			isolated cell fractions		
gene name	probe set	fold	mean S	mean S^*	fold$_{M1}$	fold$_{F1}$	fold$_{N1}$	fold$_{M2}$	fold$_{F2}$	fold$_{N2}$
dermatopontin mRNA	38059_g_at	20.8	3110.3	149.3	41.8	- 1.7	20.4	40.0	- 3.2	25.5
extracellular matrix protein 2	39673_i_at	10.6	4616.5	433.7	11.0	- 1.2	61.4	11.2	- 1.2	62.1
transforming growth factor, beta receptor III	1897_at	4.7	1781.4	381.7	**7.1**	**1.4**	3.4	**1.2**	**12.7**	1.8
interleukin 11 receptor alpha chain	496_s_at	3.3	639.8	192.5	6.0	- 1.3	- 2.4	5.8	- 1.0	- 2.7
filamin B, beta (actin binding protein 278)	38078_at	3.0	758.6	249.7	4.4	1.2	-1.2	3.7	2.3	-1.1
bone morphogenetic protein 4	40333_at	2.6	491.5	191.9	**3.3**	**1.4**	1.6	**1.3**	**5.8**	1.0
TNF receptor superfamily, member 11b	37611_at	-4.6	61.0	278.3	- 4.8	- 3.6	- 4.2	- 4.9	- 3.7	- 4.9
interleukin 11	35464_at	- 49.5	3.4	167.9	- 22.5	**- 25.3**	- 46.0	- 21.7	**- 126.7**	- 59.8
Human interleukin 15	1036_at	1.0	75.0	74.6	1.2	1.6	- 2.0	1.2	1.2	- 1.5
myosin regulatory light chain MRLC2	41187_at	1.0	2498.1	2473.5	1.1	- 1.1	1.0	1.0	- 1.0	- 1.0

Table H.1: Cell type-specific fold changes calculated from the PDF in Equation (H.4) using standard deviations obtained from expression level-based regression (see text) (fold$_{M1}$, fold$_{F1}$, fold$_{N1}$) and directly from the isolated cell fractions (fold$_{M2}$, fold$_{F2}$, fold$_{N2}$). Although the calculated cell type-specific fold changes agree in general, they can be reversed between the two methods, e.g. for the transforming growth factor, beta receptor III, and the bone morphogenetic protein 4, or can be markedly different, e.g. for interleukin 11 (numbers typed in bold face). The fold changes are based on the numerically calculated modus (most probable value) of the non-linearized PDF in Equation (H.4). The standard deviations of the fold changes (data not shown) depend on the choice of the scaling factor for the standard deviation $\hat{\sigma}_{sp}$ of the regularizing Gaussians and were below 10% for all values shown (scaling factor 0.1). Expectation values and standard deviations were numerically calculated using a closed extended integration formula with the same order as Simpson's rule [9].

References

[1] Delyon B, Juditsky A, Benveniste A: **On the relationship between identification and local tests**. Tech. Rep. 1104, IRISA Rennes Cedex, France 1997, [www.irisa.fr/sigma2/by-name/delyon.html].

[2] Fisz M: *Probability Theory and Mathematical Statistics*. Krieger Publishing Company, Malabar 1980.

[3] Gnedenko B: *Lehrbuch der Wahrscheinlichkeitstheorie*. Harri Deutsch, Thun and Frankfurt a.M. 1997.

[4] Stuart A, Ord J: *Kendall's Advanced Theory of Statistics*, Volume 1. Oxford University Press, New York 1987.

[5] Embrechts P, Klüppelberg C, Mikosch T: *Modelling extremal events*. Springer, Berlin, Heidelberg 2003.

[6] Arnett F, Edworthy S, Bloch D, McShane D, Fries J, Cooper N, Healey L, Kaplan S, Liang M, Luthra H, et al: **The American Rheumatism Association 1987 revised criteria for the classification of rheumatoid arthritis**. *Arthritis Rheum* 1988, **31**(3):315–24.

[7] Altman R, Asch E, Bloch D, Bole G, Borenstein D, Brandt K, Christy W, Cooke TD, Greenwald R, Hochberg Mea: **Development of criteria for the classification and reporting of osteoarthritis. Classification of osteoarthritis of the knee. Diagnostic and Therapeutic Criteria Committee of the American Rheumatism Association.** *Arthritis Rheum.* 1986, **29**(8):1039–1049.

[8] Zimmermann T, Kunisch E, Pfeiffer R, Hirth A, Stahl H, Sack U, Laube A, Liesaus E, Roth A, Palombo-Kinne E, Emmrich F, Kinne R: **Isolation and characterization of rheumatoid arthritis synovial fibroblasts from primary culture–primary culture cells markedly differ from fourth-passage cells**. *Arthritis Res.* 2001, **3**:72–76.

[9] Press W, Teukolsky S, Vetterling W, Flannery B: *Numerical Recipes in C*. Cambridge University Press, Cambridge 2002. [P. 134].
